# Biological Activities and In Silico Molecular Docking of an Aqueous Extract of *Nigella sativa* L. Seeds: A Focus on Antioxidant, Cytotoxic, and Diuretic Effects

**DOI:** 10.3390/ph19091418

**Published:** 2026-09-08

**Authors:** Otmane Zouirech, Rafik El-Mernissi, Mohamed Amine el Hajjaji, Marouane Takie, Mohammed Bouslamti, Abdelkrim Agour, Jawaher H. Alqahtani, Moneerah J. Alqahtani, Naoufal El Hachlafi, Joe Miantezila Basilua, Elhoussine Derwich

**Affiliations:** 1 Laboratory of Biotechnology, Conservation and Valorization of Bioresources (LBCVB), Faculty of Sciences Dhar El Mehraz, Sidi Mohamed Ben Abdellah University, Fez 30000, Morocco; otmane.zouirech@usmba.ac.ma (O.Z.); marouane.takie@usmba.ac.ma (M.T.);; 2 Bioactive and Environmental Health Laboratory, Faculty of Sciences, Moulay Ismail University, B.P. 11201, Meknes 50070, Morocco; 3 Department of Pharmacognosy, College of Pharmacy, King Saud University, P.O. Box 2457, Riyadh 11451, Saudi Arabia; jalqahtani@ksu.edu.sa; 4 Faculty of Medicine and Pharmacy, Ibn Zohr University, Guelmim 81000, Morocco; 5 Department of Biostatistics and Mathematics, Faculty of Pharmacy, Paris Cité University, 4 avenue de l’observatoire, 75006 Paris, France; joe.miantezila-basilua@u-paris.fr; 6 Unity of GC/MS and GC-FID, City of Innovation, Sidi Mohamed Ben Abdallah University, Fez 30000, Morocco

**Keywords:** *Nigella sativa*, aqueous extract, antioxidant, diuretic, cytotoxicity, ADME, phytotherapy, SDG3

## Abstract

**Background:** *Nigella sativa* L. is a medicinal plant widely recognized for its diverse pharmacological properties. This study aimed to evaluate the antioxidant, diuretic, and cytotoxic effects of an aqueous seed extract and its effects on selected biochemical parameters in rats. The pharmacokinetic potential of identified bioactive compounds was also investigated in silico. **Methods:** The aqueous extract was prepared by maceration. Antioxidant activity was assessed using DPPH, reducing power, and total antioxidant capacity (TAC) assays. We evaluated the diuretic effect in vivo in Wistar rats by measuring urinary concentrations of Na^+^, K^+^, Cl^−^, and creatinine, using furosemide as a reference drug. Cytotoxicity was assessed in splenocytes and thymocytes. We analyzed seven identified compounds in silico for physicochemical properties, Lipinski compliance, intestinal absorption, cytochrome P_450_ inhibition, and blood–brain barrier permeability. **Results:** The extract showed significant antioxidant activity, with a DPPH IC_50_ of 0.254 ± 0.002 mg/mL and a TAC of 125.01 ± 4.220 mg AAE/g. It significantly increased urinary electrolyte and urinary concentration, indicating a diuretic effect lower than that of furosemide. Plasma analyses showed decreased electrolyte concentrations and increased creatinine levels. Cell viability exceeded 90%, indicating no significant cytotoxicity. Most compounds complied with Lipinski’s rule of five and showed favorable predicted intestinal absorption, with limited cytochrome P_450_ inhibition. Several compounds were predicted to cross the blood–brain barrier. **Conclusions:** The aqueous extract of *N. sativa* demonstrated antioxidant and diuretic activities without significant cytotoxicity. These findings support its pharmacological potential and warrant further investigation of its mechanisms of action and therapeutic relevance.

## 1. Introduction

Since antiquity, medicinal plants have held a central place in traditional healing practices across the world. Far from being relegated to the status of ancestral remedies, they are now attracting renewed interest within the scientific community, particularly due to the therapeutic limitations, increasing drug resistance, and sometimes severe side effects associated with synthetic treatments [1]. In this context, the exploration of plant extracts, especially those obtained through gentle methods such as aqueous extraction, represents a promising avenue for the discovery of new bioactive molecules with therapeutic potential.

Among the plant species that have drawn the attention of researchers, *Nigella sativa* L., also known as black seed or “*habba sawda*”, stands out due to the richness of its chemical composition and the diversity of its traditional medicinal uses [2]. It is widely used in several systems of traditional medicine, including Arab-Islamic, Ayurvedic, African, and Mediterranean medicine, to treat a wide range of ailments, from digestive and respiratory disorders to infections, inflammation, and metabolic imbalances, as well as to support immune function [3,4].

From a scientific perspective, the growing interest in *Nigella sativa* is largely attributed to the identification of numerous bioactive compounds, such as thymoquinone, flavonoids, phenolic acids, alkaloids, and saponins, which have demonstrated various pharmacological properties including antioxidant, anti-inflammatory, antimicrobial, and anticancer activities [5,6,7]. However, while the essential oils and lipophilic extracts of this plant have been extensively studied, the aqueous extract, more closely aligned with the galenic forms traditionally used (infusions, decoctions), remains relatively underexplored, despite its advantages of being non-toxic, cost-effective, and easily applicable on a large scale in medicinal or nutritional contexts [8,9].

The use of medicinal plants as diuretics is an ancient and well-documented practice, which is currently attracting renewed interest due to the side effects sometimes associated with synthetic diuretics [10]. Numerous preclinical studies now confirm the diuretic potential of these plants [10,11,12]. For instance, extracts of *Tribulus terrestris*, *Hedyotis scandens*, or *Silybum marianum* have shown a significant increase in urine volume and sodium excretion in animal models, with effects sometimes comparable to those of a reference drug such as furosemide [10,13]. However, most of the evidence remains preclinical, and rigorous clinical trials are still needed to fully validate their efficacy and safety in humans, especially since drug interactions are possible.

This study aims to contribute to a better understanding of the properties of the aqueous extract of *Nigella sativa* seeds. It first seeks to characterize its phytochemical composition, particularly in terms of polyphenols and flavonoids, using chromatographic and spectrophotometric analyses. The study also evaluates its antioxidant activity, a key parameter in the prevention of oxidative stress, a mechanism involved in numerous degenerative and metabolic diseases. Furthermore, it investigates the extract’s potential diuretic effect, in relation to its traditional use in renal and urinary disorders, and its impact on the regulation of hydro-sodium homeostasis. Finally, an assessment of its cytotoxicity is conducted through in vitro tests on primary rabbit splenocytes and thymocytes to ensure its safety, thereby providing insights into its potential applications in the pharmaceutical, nutraceutical, or therapeutic fields.

This integrated approach aims to scientifically valorize a medicinal plant of interest and to strengthen the connections between traditional knowledge and contemporary research.

## 2. Results

### 2.1. Quantitative Analysis of Phytochemical Compounds

The extraction yield obtained for the aqueous extract of *N. sativa* seeds was 17 ± 1.22%. This relatively high yield is due to the seeds’ high content of hydrophilic compounds, such as phenols, flavonoids, sugars, and other water-soluble secondary metabolites. Phytochemical analyses of the aqueous extract from *N. sativa* seeds revealed significant concentrations of bioactive compounds, particularly polyphenols, flavonoids, and total carbohydrates. These results are summarized in Table 1.

#### 2.1.1. Determination of Total Phenolic Content (TPC)

Quantitative analysis of total polyphenols in the aqueous extract of *N. sativa* seeds showed a concentration of 22.88 ± 1.12 mg GAE/g DW (Table 1). This significant content of water-soluble phenolic compounds reflects the richness of the extract in bioactive substances known for their antioxidant properties. Polyphenols play a crucial role in neutralizing free radicals, thereby helping prevent oxidative stress and protecting cells from oxidative damage.

#### 2.1.2. Determination of Total Flavonoid Content (TFC)

The total flavonoid content of the aqueous extract of *N. sativa* seeds was found to be 19.14 ± 1.31 mg QE/g DW (Table 1). This value indicates a noteworthy presence of water-soluble flavonoids, compounds known for their antioxidant, anti-inflammatory, and antimicrobial properties. Flavonoids are essential in neutralizing free radicals, thus contributing to cellular protection against oxidative damage.

#### 2.1.3. Total Carbohydrate Content

The total carbohydrate content of the aqueous extract of *N. sativa* was quantified at 5.81± 0.04 g GluE/100 g D.W. (Table 1). This result indicates a moderate carbohydrate concentration, which is typical for aqueous extracts of medicinal plants. The total carbohydrate content reinforces the multifunctional potential of the aqueous extract of *N. sativa*, particularly from a biological perspective.

The carbohydrates present do not merely serve as energy substrates: they may also play important functional roles, acting as prebiotics, modulating immune responses, or participating in cellular signaling. These properties give them particular interest in the fields of health and nutrition.

Moreover, the presence of carbohydrates in the extract can influence physicochemical properties such as viscosity or colloidal stability—key criteria in the development of plant-based formulations for pharmaceutical or nutraceutical applications.

#### 2.1.4. HPLC Analysis

**Figure 1:**
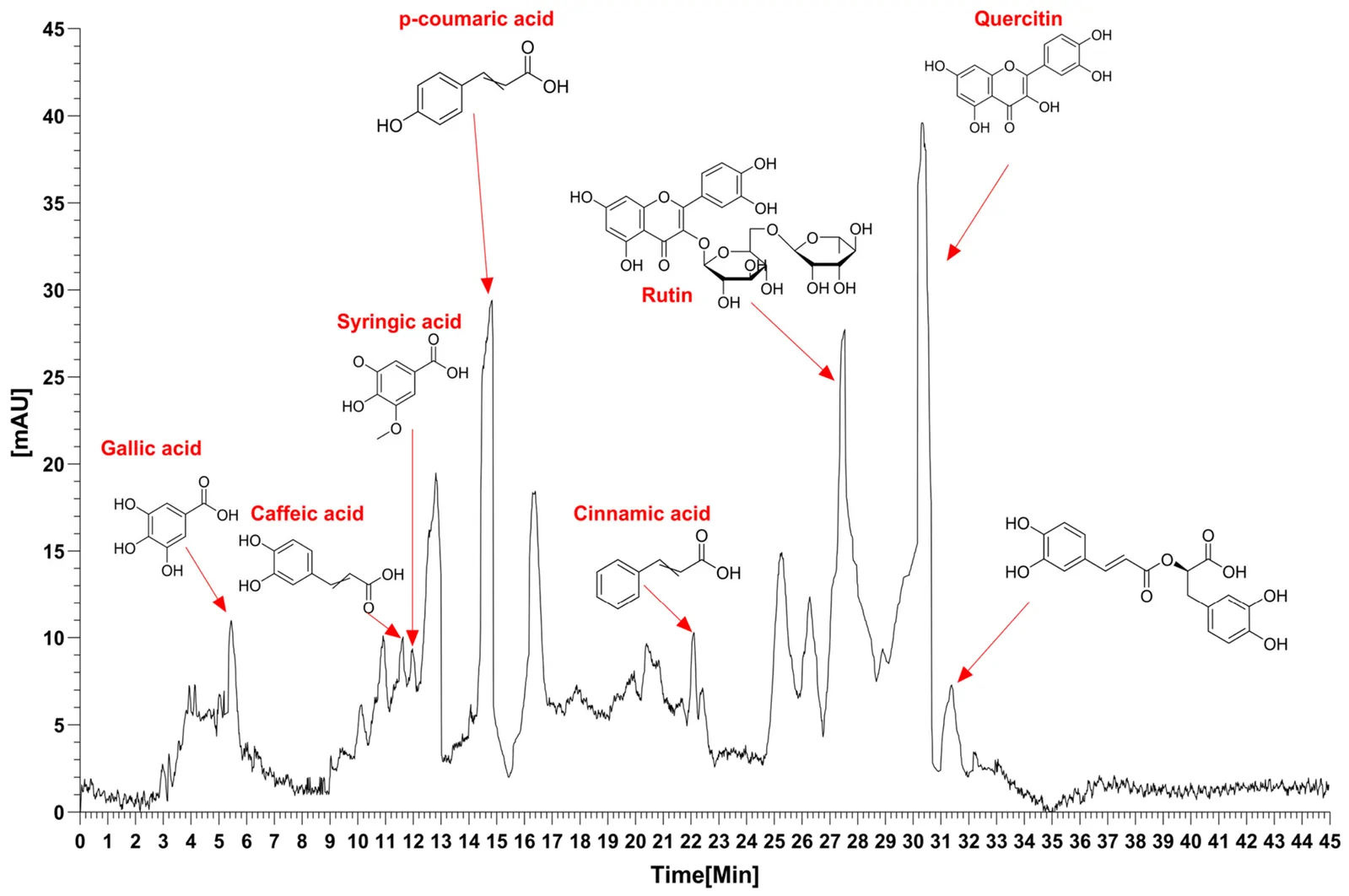
HPLC-DAD chromatogram of the phenolic compounds tentatively assigned by HPLC-DAD.

The HPLC-DAD analysis of the chromatogram presented in Figure 1 allowed the identification and quantification of eight distinct phenolic compounds, with retention times ranging from 5.45 to 31.60 min. The chromatographic profile is dominated by quercetin, which represents the largest fraction with 29.58% of the total relative peak area, followed by rutin (14.06%) and p-coumaric acid (13.19%). The other identified compounds are present in lower proportions, namely gallic acid (5.77%), rosmarinic acid (4.92%), and cinnamic acid (2.33%), while caffeic and syringic acids are only detected in trace amounts, with 0.49% and 0.31% of the relative peak areas, respectively (Table 2). The chromatographic separation proves satisfactory for all peaks, confirming the richness of the sample in phenolic compounds, with a marked predominance of flavonols (quercetin and rutin) over simple phenolic acids. The resulting profile reveals a diversity of phenolic and flavonoid compounds, reflecting the antioxidant potential of the extract. The notable presence of quercetin and rutin, two well-known flavonoids with pharmacological effects, reinforces the therapeutic value of *N. sativa*.

### 2.2. Antioxidant Activity

#### 2.2.1. DPPH Assay

**Figure 2:**
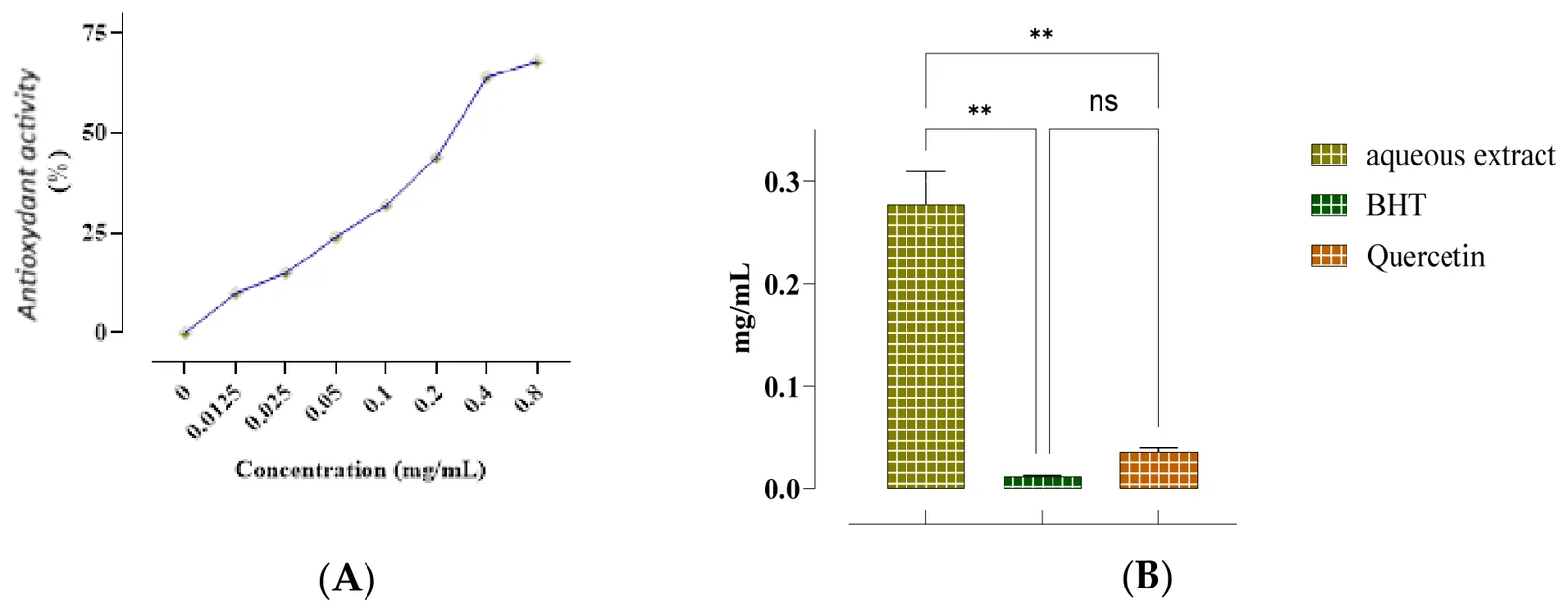
(**A**) DPPH antioxidant activity of the aqueous extract of *N. sativa*; (**B**) IC_50_ values for the extract, BHT and quercetin. The values shown are the means of three independent experiments. Values are expressed as mean ± standard deviation (SD). ** *p* < 0.01 compared to the control group; ns: not significant compared to the control group.

The antioxidant activity of the aqueous extract of *N. sativa* was evaluated using the DPPH free radical scavenging method. Figure 2 illustrates the progressive increase in the percentage of DPPH inhibition with increasing concentrations of the extract. At a concentration of 0.012 mg/mL, the extract shows moderate inhibition of 10%, which gradually increases to reach 68% at the highest concentration tested (0.8 mg/mL). This dose-dependent profile reflects an increasing antioxidant potential with concentration. The determination of the IC_50_ allowed for a more accurate comparison of the antioxidant efficacy of the extract against two standard antioxidants, BHT (butylated hydroxytoluene) and quercetin (Table 2). The aqueous extract of *N. sativa* exhibits an IC_50_ of 0.254 ± 0.002 mg/mL, indicating a notable antioxidant activity, although significantly lower than that of BHT (0.012 ± 0.001 mg/mL) and quercetin (0.035 ± 0.040 mg/mL).

#### 2.2.2. FRAP Assay

**Figure 3:**
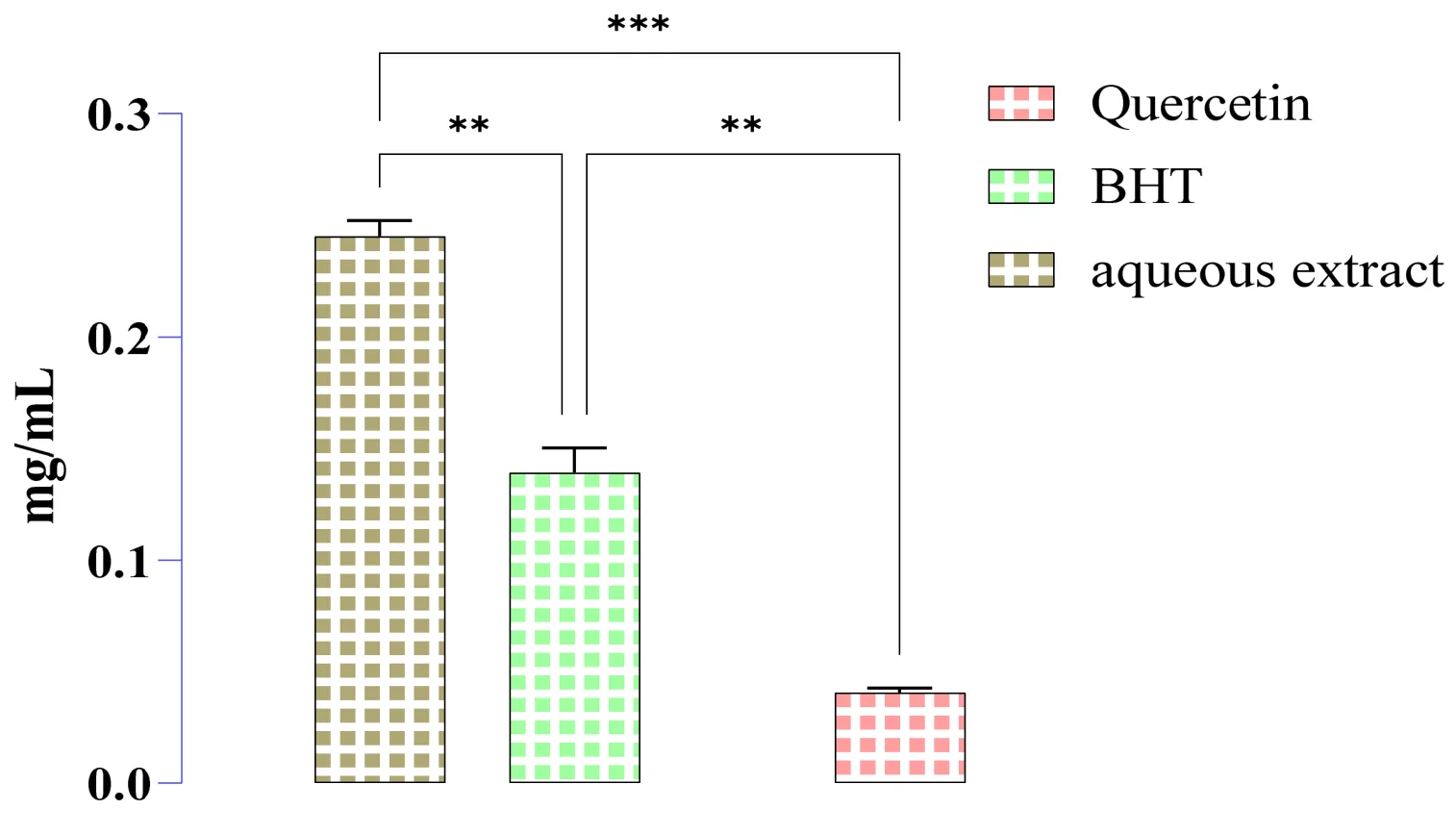
Antioxidant power of the aqueous extract of *N. sativa*, compared to BHT and quercetin. Values are expressed as mean ± standard deviation (SD). ** *p* < 0.01 compared to the control group; *** *p* < 0.001 compared to the control group.

The antioxidant power of the aqueous extract of *N. sativa* was also assessed based on EC_50_ values, in comparison with those of BHT and quercetin (Figure 3). EC_50_ represents the concentration of antioxidant required to achieve 50% of the maximum antioxidant effect (Table 3).

The aqueous extract showed an average EC_50_ of 0.247 ± 0.012 mg/mL, indicating moderate efficacy. In comparison, the EC_50_ values obtained for the reference antioxidants were significantly lower: 0.139 ± 0.011 mg/mL for BHT and 0.041 ± 0.020 mg/mL for quercetin, confirming their strong ability to neutralize free radicals at much lower concentrations. These results confirm that the aqueous extract of *N. sativa* possesses noteworthy antioxidant activity, although it is less potent than that of standard antioxidants. This activity may be attributed to the presence of phenolic compounds and other water-soluble bioactive substances.

#### 2.2.3. TAC Assay

**Figure 4:**
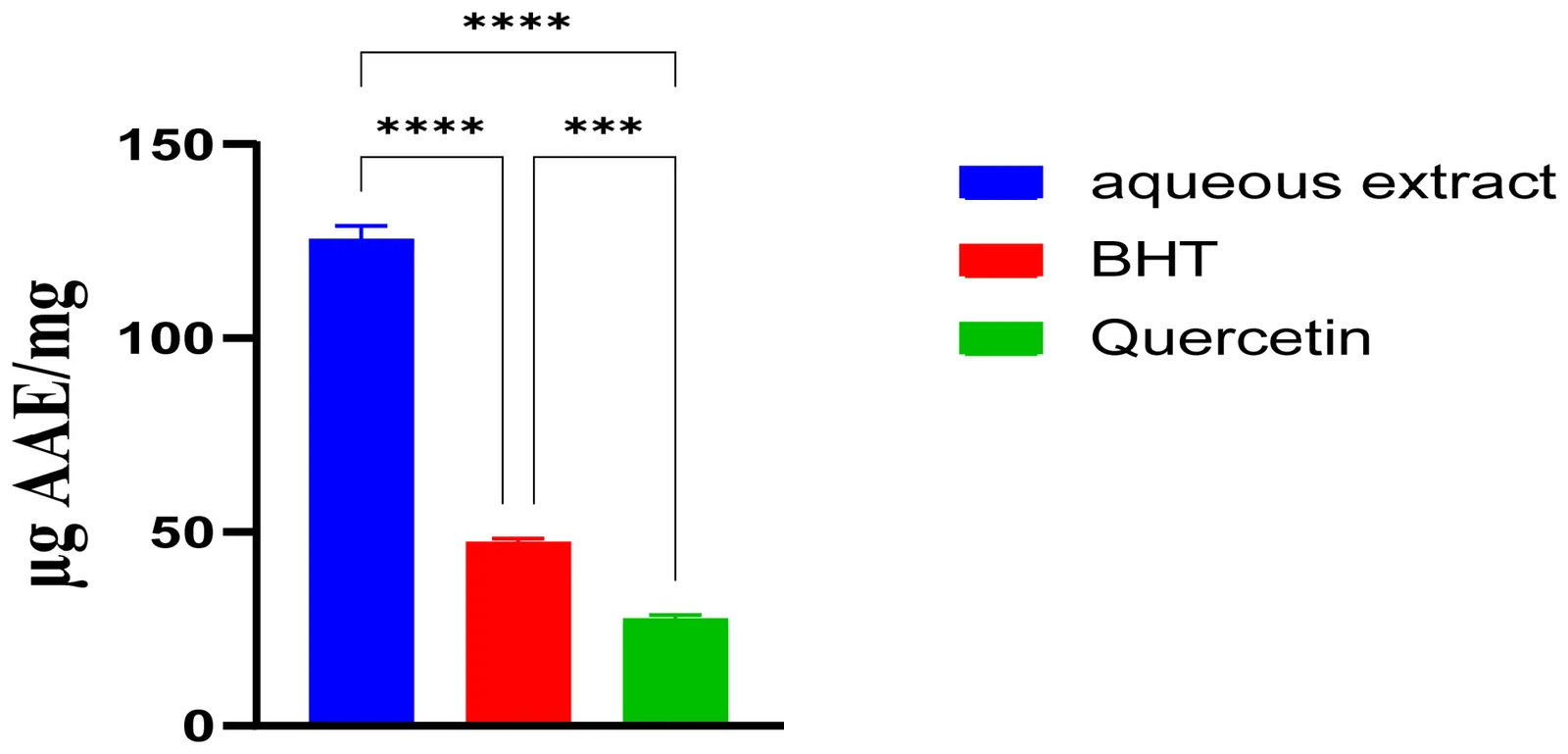
Antioxidant capacity of the aqueous extract of *N. sativa*, compared to BHT and quercetin. Values are expressed as mean ± standard deviation (SD). *** *p* < 0.001 compared to the control group; **** *p* < 0.0001 compared to the control group.

The total antioxidant capacity of the aqueous extract of *N. sativa* was evaluated and compared with that of standard antioxidants BHT and quercetin (Figure 4). The results, expressed in µg ascorbic acid equivalent/mg of sample, revealed a significant antioxidant activity of the extract. The aqueous extract showed an average TAC of 125.01 ± 4.22 mg AAE/g, indicating a high antioxidant potential. In comparison, BHT and quercetin showed values of 47.28 ± 1.050 mg AAE/g and 27.81 ± 0.981 mg AAE/g, respectively (Table 2). These results indicate that the aqueous extract of *N. sativa* exhibits a higher overall free radical reducing capacity than the two reference antioxidants in this test.

### 2.3. Cytotoxic Aspect

**Figure 5:**
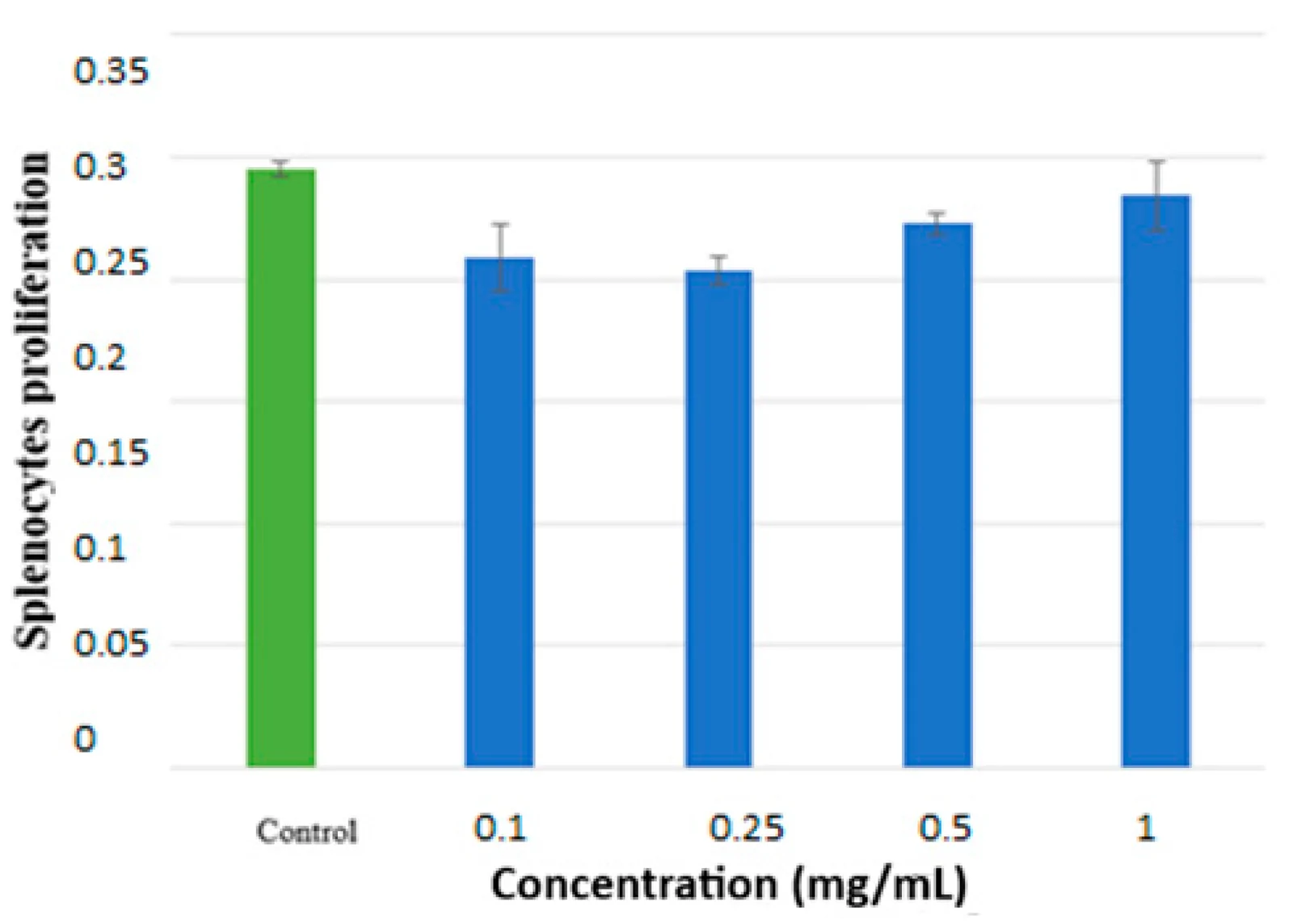
Effect of the aqueous extract of *N. sativa* on splenocyte proliferation. Values represent the mean ± SD from six animals (*n* = 6). Error bars on each histogram indicate the standard deviation.

The toxicity of the aqueous extract of *N. sativa* was evaluated using the MTT assay on primary immune cells derived from the spleen and thymus, exposed to concentrations ranging from 0.1 to 1 mg/mL. The results were compared to those of the untreated control under the same experimental conditions (Figure 5).

In splenocytes, the aqueous extract of *N. sativa* did not cause any significant variation in cell viability. At the highest concentrations tested (0.5 and 1 mg/mL), the viability rates were 90.90% and 95.52%, respectively—values that fall within the range considered normal, thus indicating the absence of toxicity of the extract at these concentrations.

**Figure 6:**
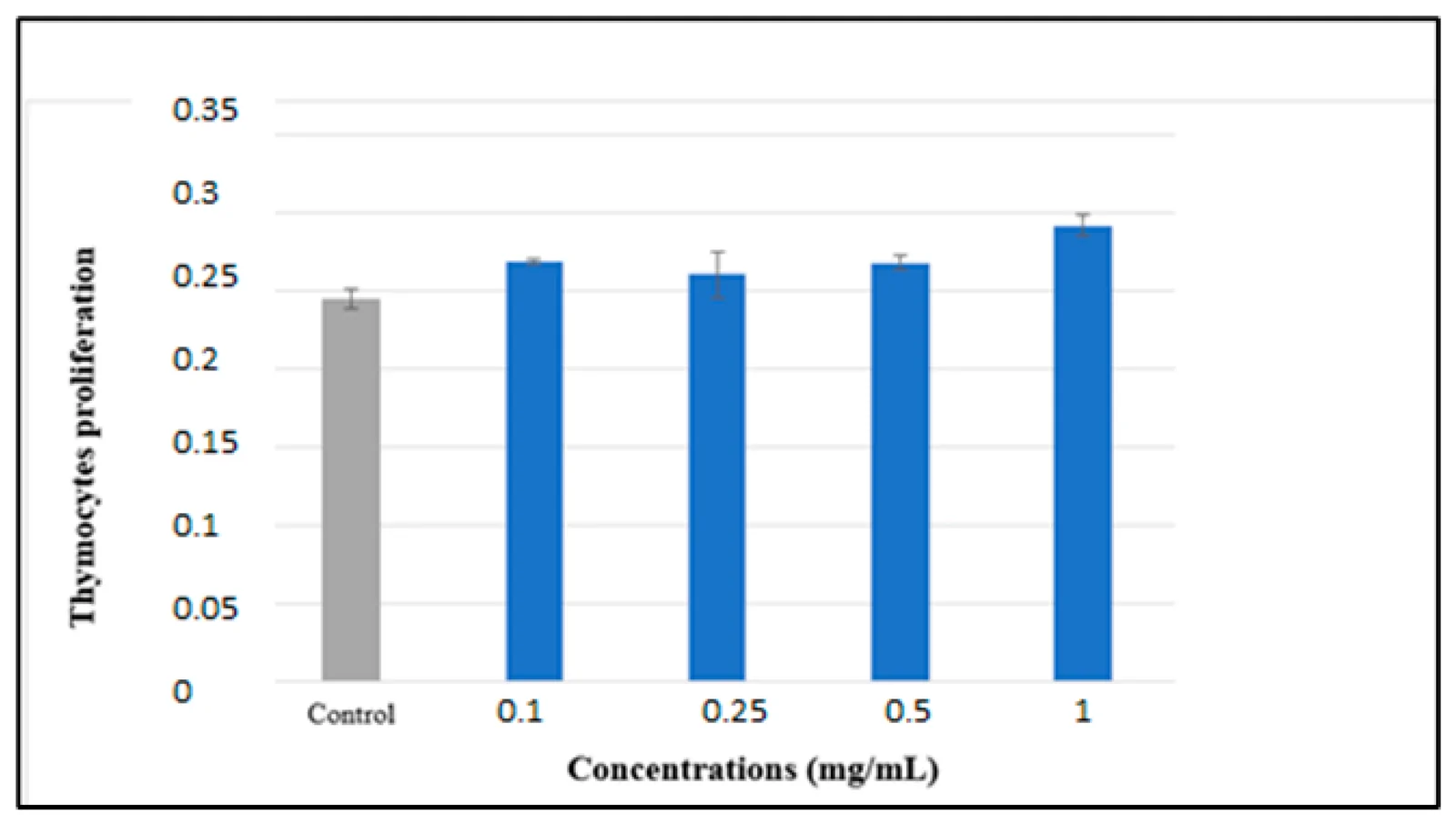
Effect of the aqueous extract of *N. sativa* on thymocyte proliferation. Values represent the mean ± SD from six animals (*n* = 6). Error bars on each histogram indicate the standard deviation.

Exposure of thymocytes to the aqueous extract of *N. sativa* resulted in a consistent increase in their viability, with rates ranging from 106.48% to 119.39% at all tested concentrations (Figure 6). This observation indicates a very good tolerance of these cells to the extract and potentially suggests a stimulation of their metabolic activity.

Overall, the *N. sativa* extract did not exhibit significant cytotoxicity toward splenocytes. However, it induced a slight, non-significant increase in metabolic activity, which was more pronounced in thymocytes. Viability values maintained near or above 100% reflect the preservation or enhancement of mitochondrial metabolic activity, confirming that these aqueous extracts are non-toxic, well tolerated, and highly biocompatible. The favorable profile observed may be attributed to the hydrophilic nature of the extract’s constituents, which is often associated with better biocompatibility. Under the experimental conditions used, the aqueous extract showed no significant cytotoxic effects on primary splenocytes and thymocytes at the tested concentrations and exposure period.

### 2.4. Diuretic Activity of the Aqueous Extract of N. sativa

#### 2.4.1. Effect of *N. sativa* Extract and Furosemide on Urine Volume

The volume of urine was measured in graduated cylinders on days 1, 7, and 15 of treatment (day 1 corresponding to 24 h after the first gavage), and the results increased gradually in all three groups during the study, with the animals receiving furosemide and *N. sativa* showing the greatest increase in urine volume (Table 4). Urine output was already significantly increased in the furosemide (7.05 ± 0.06 mL) and *N. sativa* (8.55 ± 0.06 mL) groups relative to control (3.75 ± 0.06 mL) after the first dose (day 1), consistent with the rapid onset of action of furosemide, and continued to increase progressively over the treatment period. By day 15, urine volume reached 13.20 ± 1.15 mL in the furosemide group and 13.67 ± 0.58 mL in the *N. sativa* group, compared with 4.60 ± 0.69 mL in the control group, corresponding to diuretic indices of 2.87 and 2.97, respectively. These results indicate that the aqueous extract of *N. sativa* produced a diuretic effect on a volume basis that was comparable to, and at day 15 marginally exceeded, that of the reference drug furosemide.

#### 2.4.2. Impact of the Aqueous Extract of *N. sativa* on Urinary Electrolytes and Creatinine

**Figure 7:**
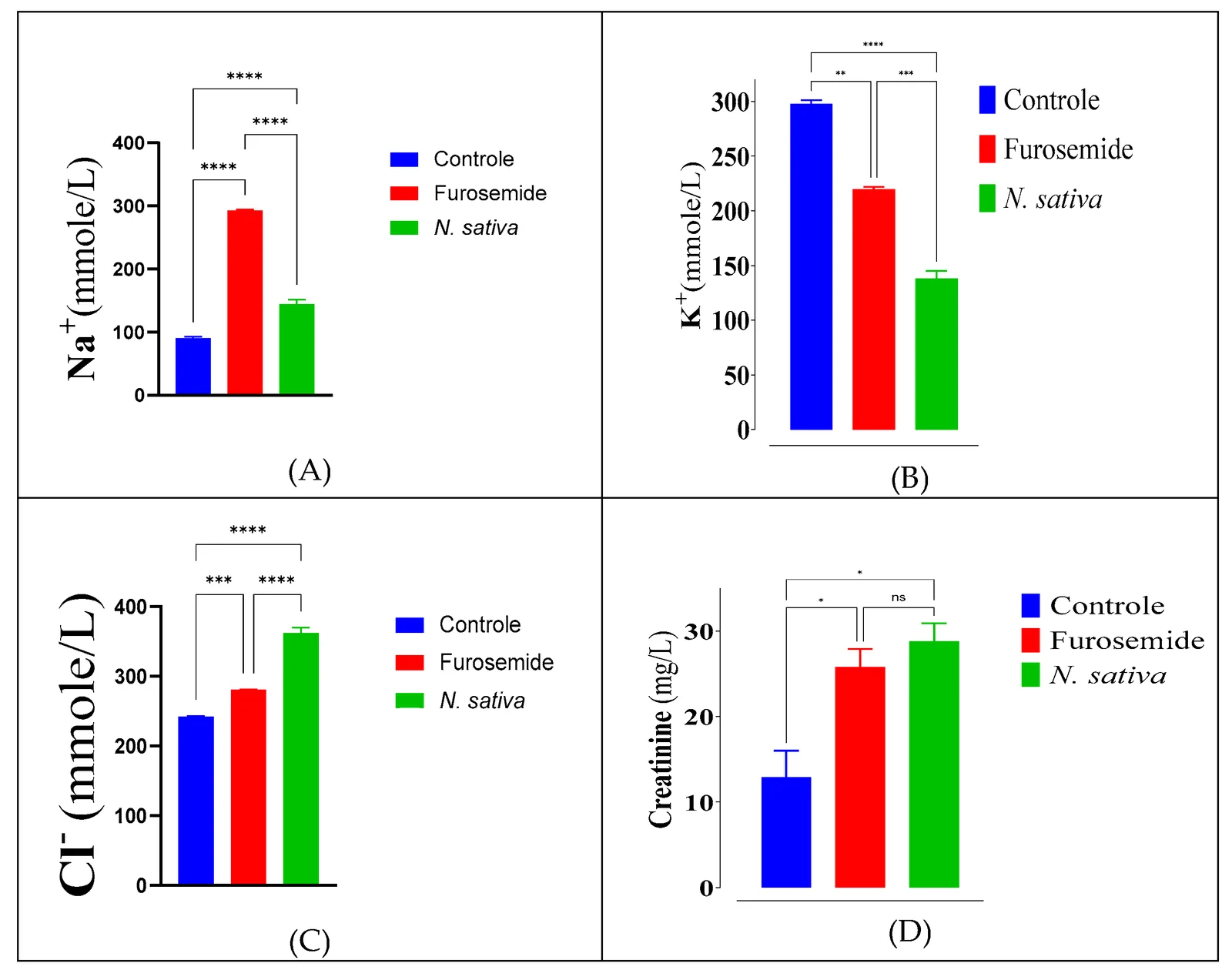
Effects of treatment with *N. sativa* aqueous extract (300 mg/kg) and furosemide (10 mg/kg) on urinary electrolyte and creatinine levels on day 15 of experimentation in rats. (**A**) Urinary sodium (Na^+^), (**B**) Urinary potassium (K^+^), (**C**) Urinary chloride (Cl^−^), (**D**) Urinary creatinine. Values are expressed as mean ± standard deviation (SD). * *p* < 0.1 compared to the control group; ** *p* < 0.01 compared to the control group; *** *p* < 0.001 compared to the control group; **** *p* < 0.0001 compared to the control group. ns: not significant compared to the control group.

The effects of the aqueous extract of *N. sativa* and furosemide on the urinary concentrations of electrolytes (Na^+^, K^+^, Cl^−^) and creatinine were evaluated and are presented in Figure 7. Compared to the control group, treatment with both furosemide and the aqueous extract induced a significant increase (*p* < 0.001) in the urinary concentrations of sodium, potassium, chloride, and creatinine.

Furosemide showed strong diuretic activity, with sodium excretion reaching 292.5 ± 2.12 mmol/L, potassium 219.5 ± 2.12 mmol/L, chloride 281.15 ± 0.21 mmol/L, and urinary creatinine concentration at 25.76 ± 2.12 mg/L. The aqueous extract of *N. sativa*, at a dose of 300 mg/kg, also demonstrated notable diuretic activity, although moderate compared to furosemide, by increasing sodium excretion to 144.5 ± 7.70 mmol/L, potassium to 138.0 ± 6.97 mmol/L, chloride to 362.0 ± 8.48 mmol/L, and creatinine to 28.0 ± 2.12 mg/L.

#### 2.4.3. Effect of *N. sativa* Aqueous Extract on Plasma Electrolytes and Creatinine

**Figure 8:**
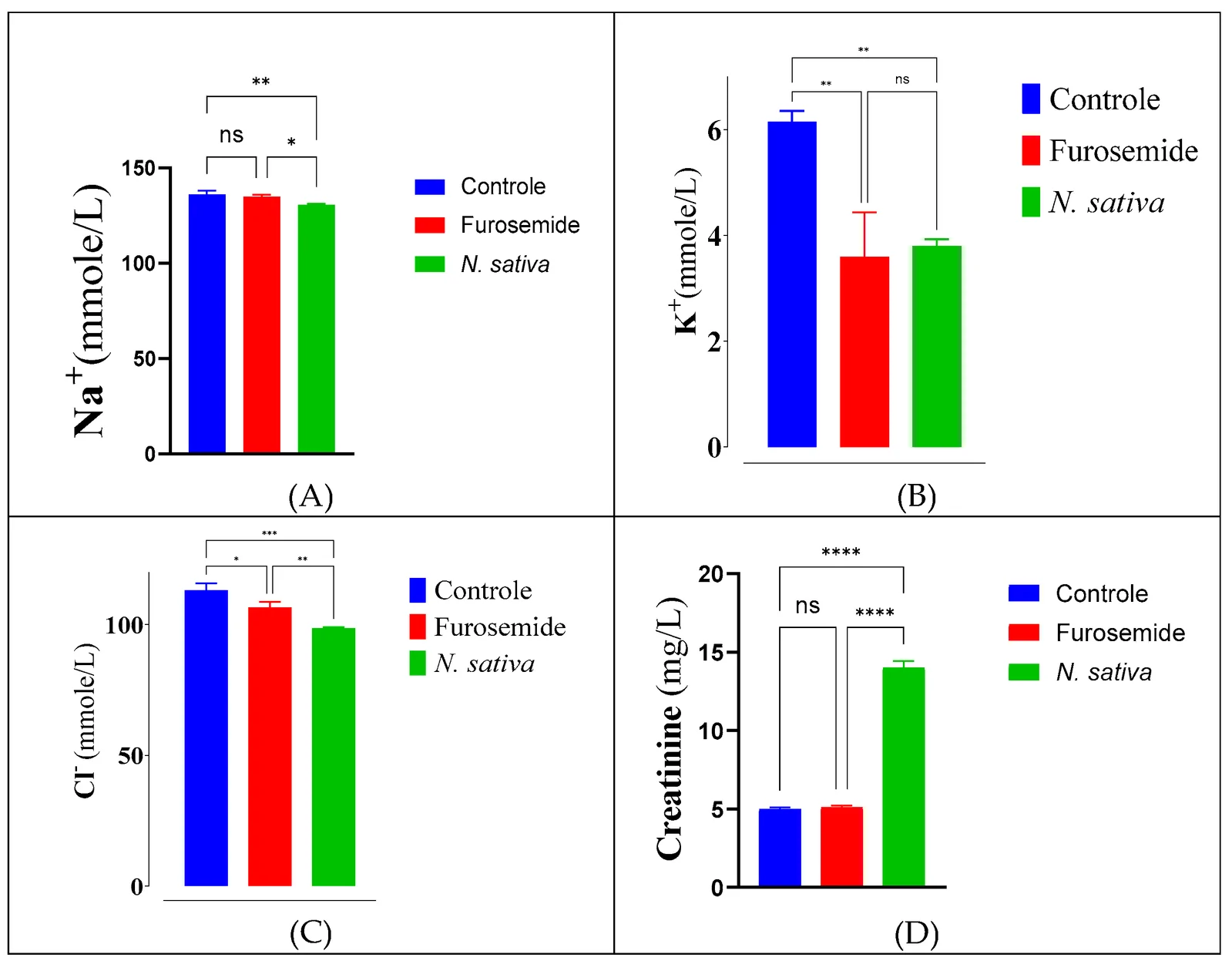
Effects of treatment with the aqueous extract of *N. sativa* (300 mg/kg) and furosemide (10 mg/kg) on plasma electrolyte and creatinine levels on day 15 of experimentation in rats. (**A**) Plasma sodium (Na^+^), (**B**) Plasma potassium (K^+^), (**C**) Plasma chloride (Cl^−^), (**D**) Plasma creatinine. Values are expressed as mean ± standard deviation (SD). * *p* < 0.1 compared to the control group; ** *p* < 0.01 compared to the control group; *** *p* < 0.001 compared to the control group; **** *p* < 0.0001 compared to the control group. ns: not significant compared to the control group.

Daily oral administration of *N. sativa* aqueous extract at 300 mg/kg for 15 days resulted in significant alterations in plasma biochemical parameters in rats compared with the control and furosemide-treated groups (Figure 8). The extract decreased plasma sodium (130.50 ± 0.70 mmol/L) and chloride (98.5 ± 0.53 mmol/L) levels compared with the control group values (136 ± 2.12 mmol/L for Na^+^ and 113 ± 2.82 mmol/L for Cl^−^). Similarly, moderate hypokalemia was observed (3.805 ± 0.134 mmol/L) compared with the control group (6.15 ± 0.21 mmol/L), consistent with the expected diuretic effect.

The *N. sativa* group exhibited a significant rise in plasma creatinine concentration to 14.00 ± 0.00 mg/L, while the control group and the animals treated with furosemide had plasma creatinine concentrations of 5.0 ± 0.00 mg/L and 5.1 mg/L, respectively. If there is no calculated creatinine clearance and urinary biomarkers (e.g., BUN/urea) and/or renal histology data, this is best interpreted as a biochemical change with an uncertain physiologic significance that requires further evaluation.

#### 2.4.4. Molecular Modeling

**Figure 9:**
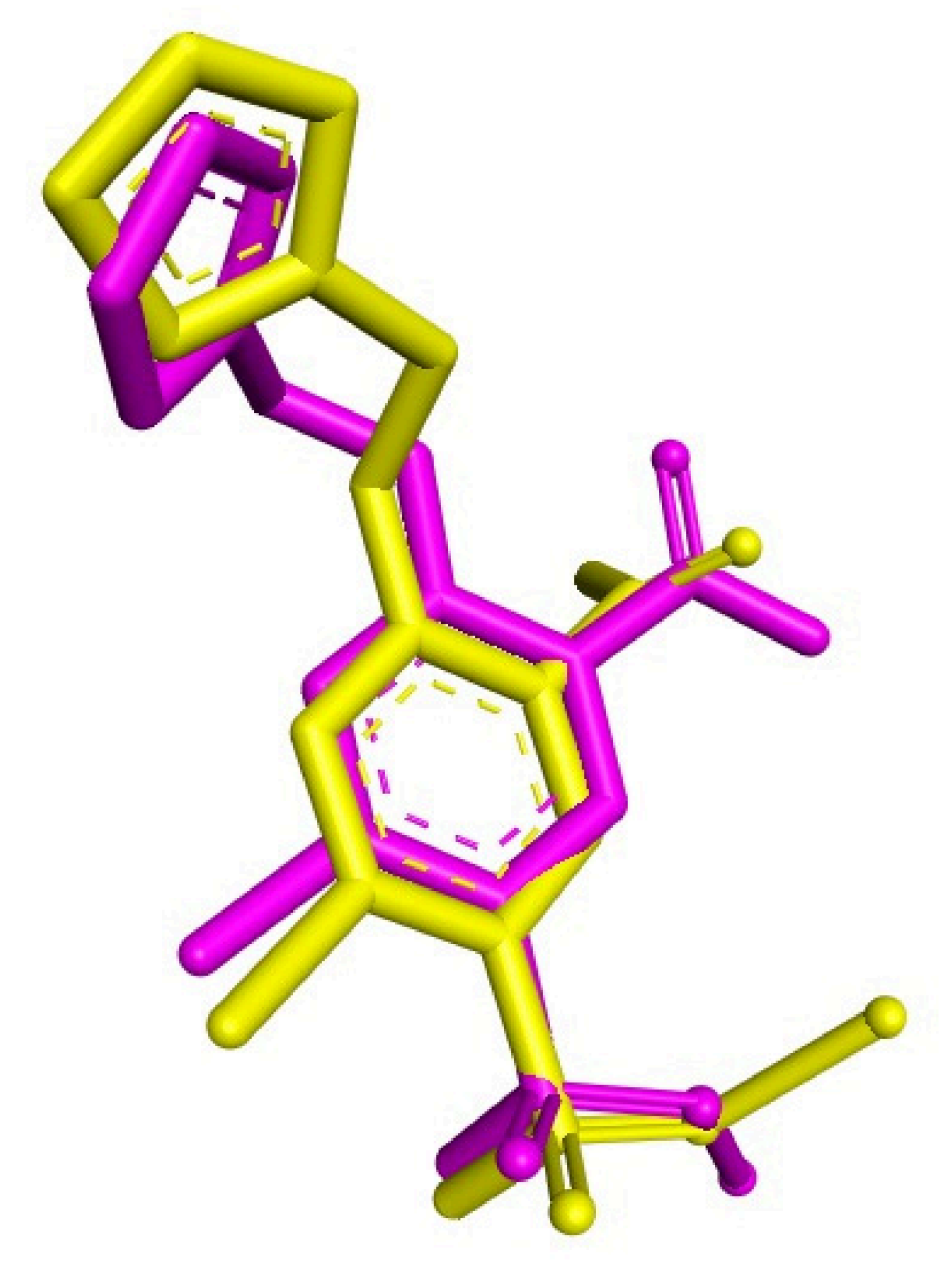
Superposition of the crystallographic and redocked conformations of furosemide in the active site of carbonic anhydrase II (PDB ID: 1Z9Y), showing an RMSD of 2.39 Å. Pink and yellow dashed lines indicate [e.g., hydrogen-bonding interactions between the ligand and key active-site residues/close contacts between the two superimposed poses/coordination to the catalytic zinc ion], respectively.

The docking protocol was validated by redocking furosemide, a sulfonamide inhibitor, into the active site of carbonic anhydrase II (PDB ID: 1Z9Y) using the same docking parameters applied to the tested compounds. The redocked ligand exhibited a high degree of structural agreement with its experimentally determined crystallographic conformation, yielding an RMSD value of 2.39 Å. This value falls within the generally accepted range for validating molecular docking protocols, supporting the reliability and reproducibility of the docking procedure employed in this study (Figure 9).

**Figure 10:**
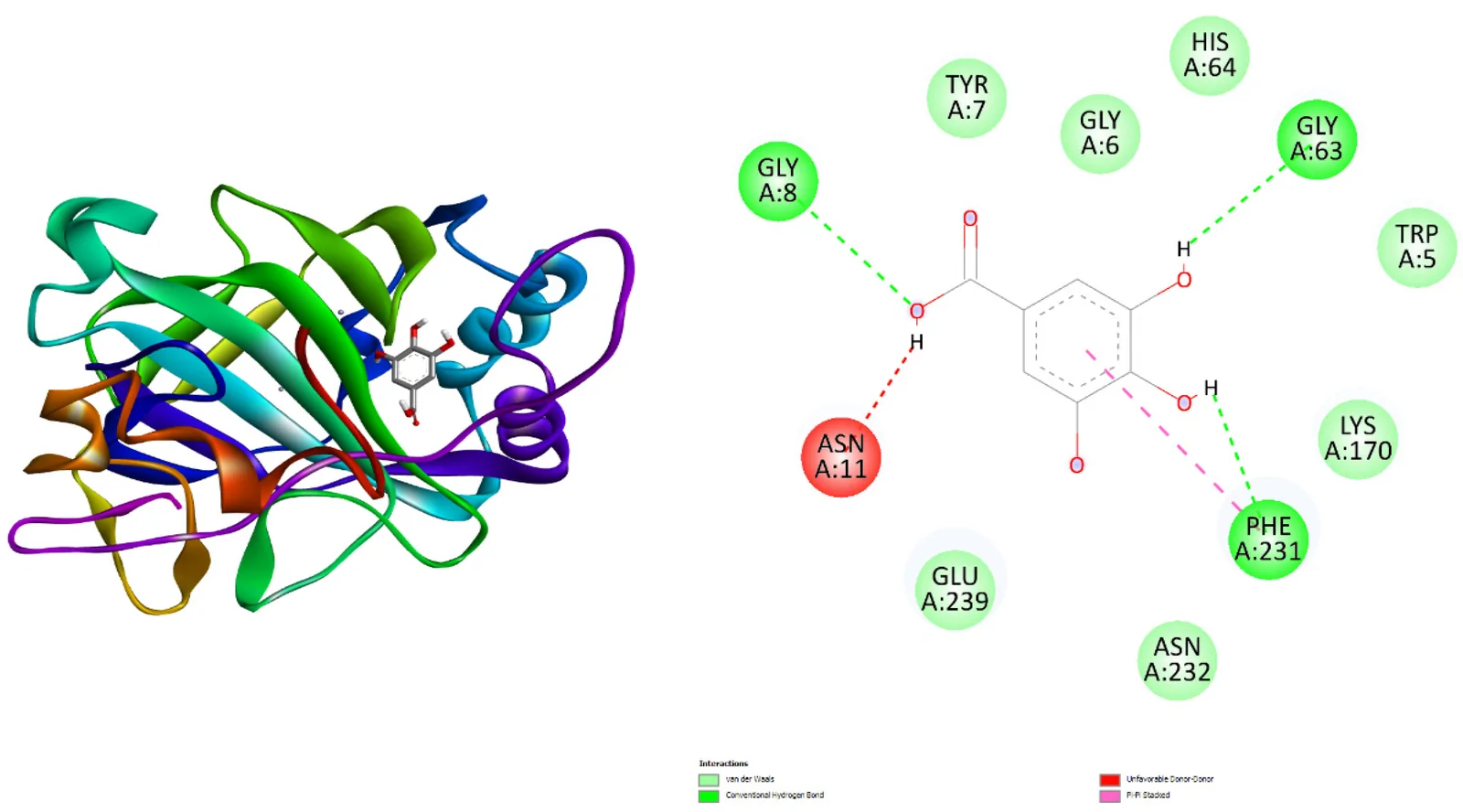
2D and 3D visualization of the docking results of gallic acid in the active site of carbonic anhydrase.

**Figure 11:**
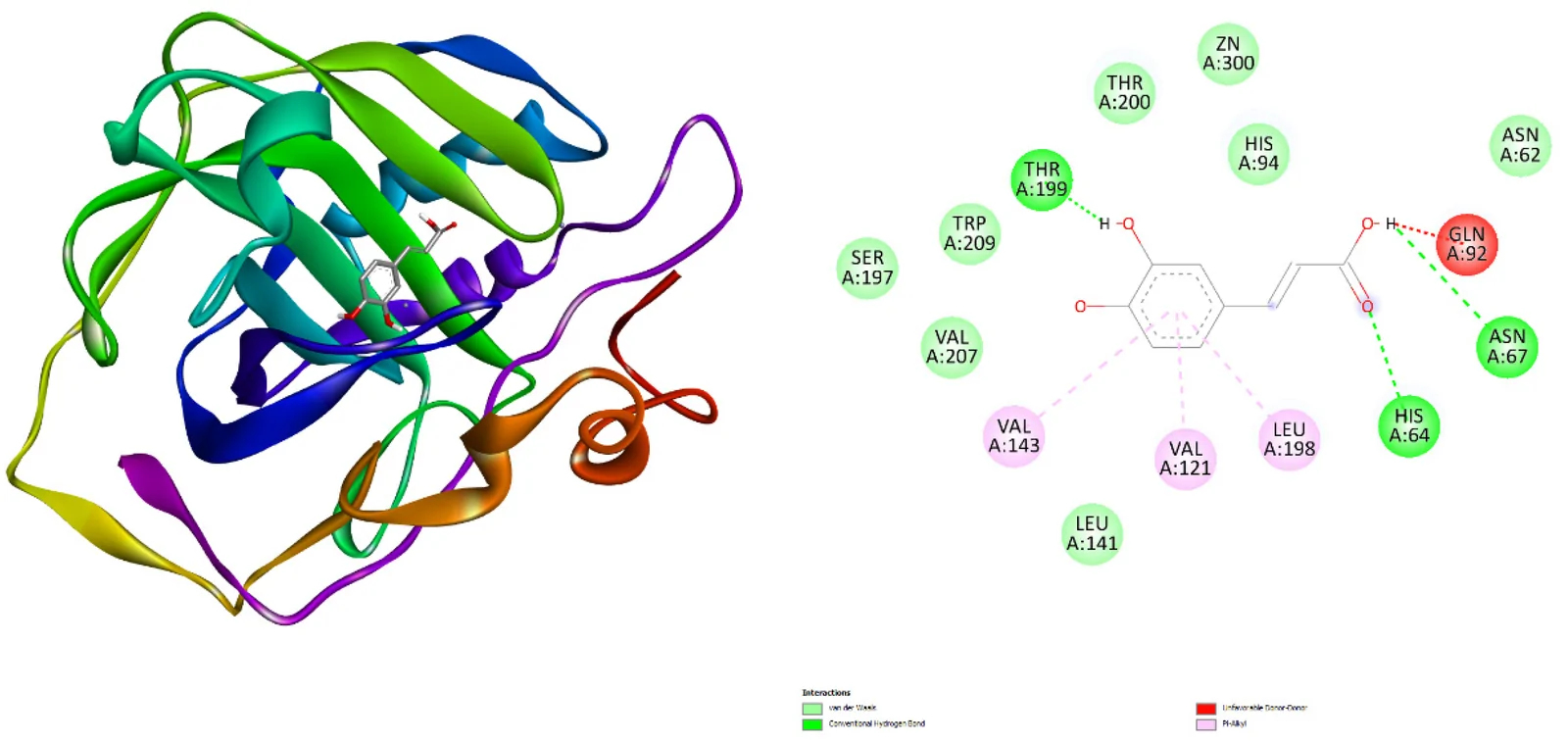
2D and 3D visualization of the docking results of caffeic acid in the active site of carbonic anhydrase.

**Figure 12:**
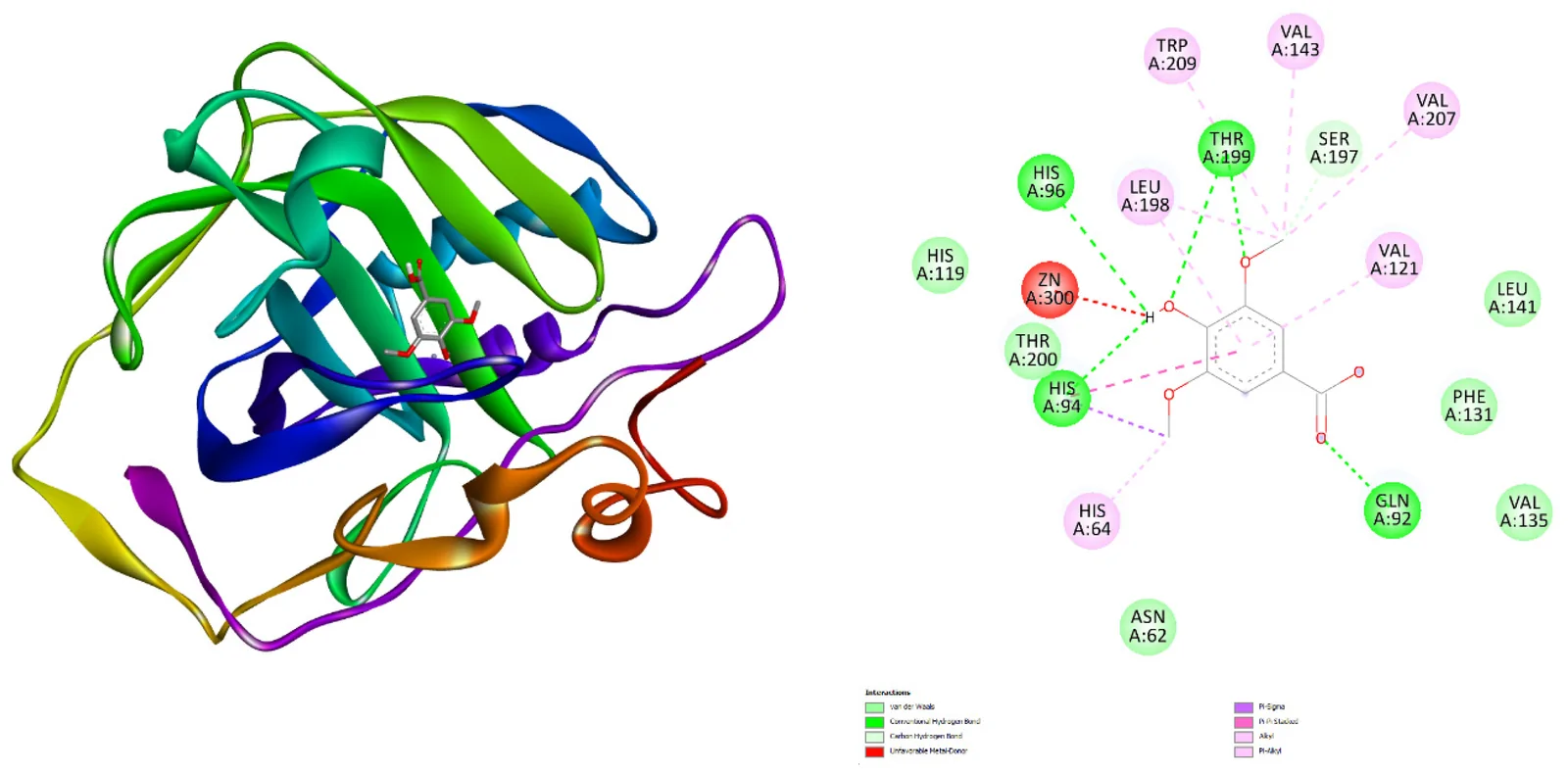
2D and 3D visualization of the docking results of syringic acid in the active site of carbonic anhydrase.

**Figure 13:**
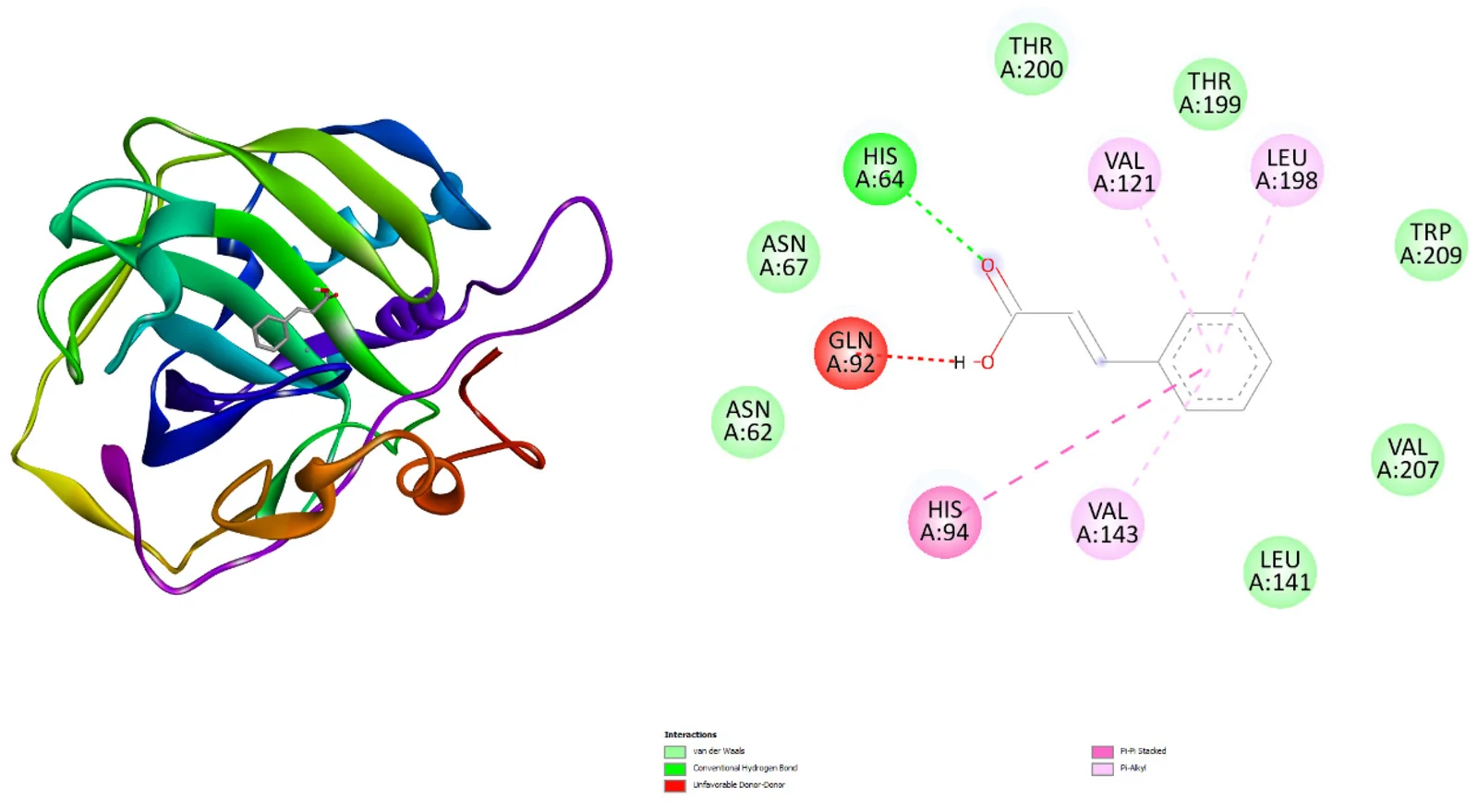
2D and 3D visualization of the docking results of cinnamic acid in the active site of carbonic anhydrase.

**Figure 14:**
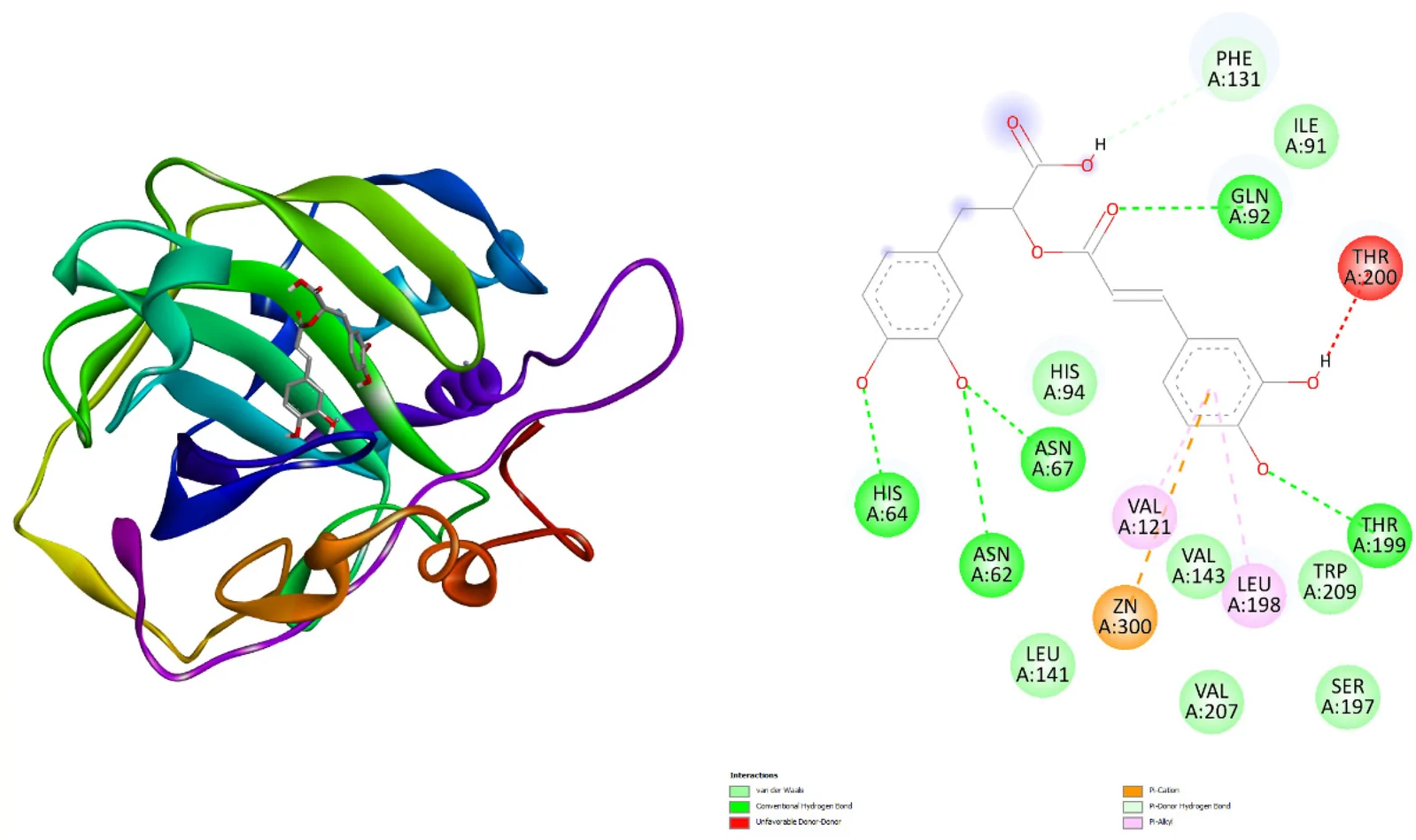
2D and 3D visualization of the docking results of rosmarinic acid in the active site of carbonic anhydrase.

**Figure 15:**
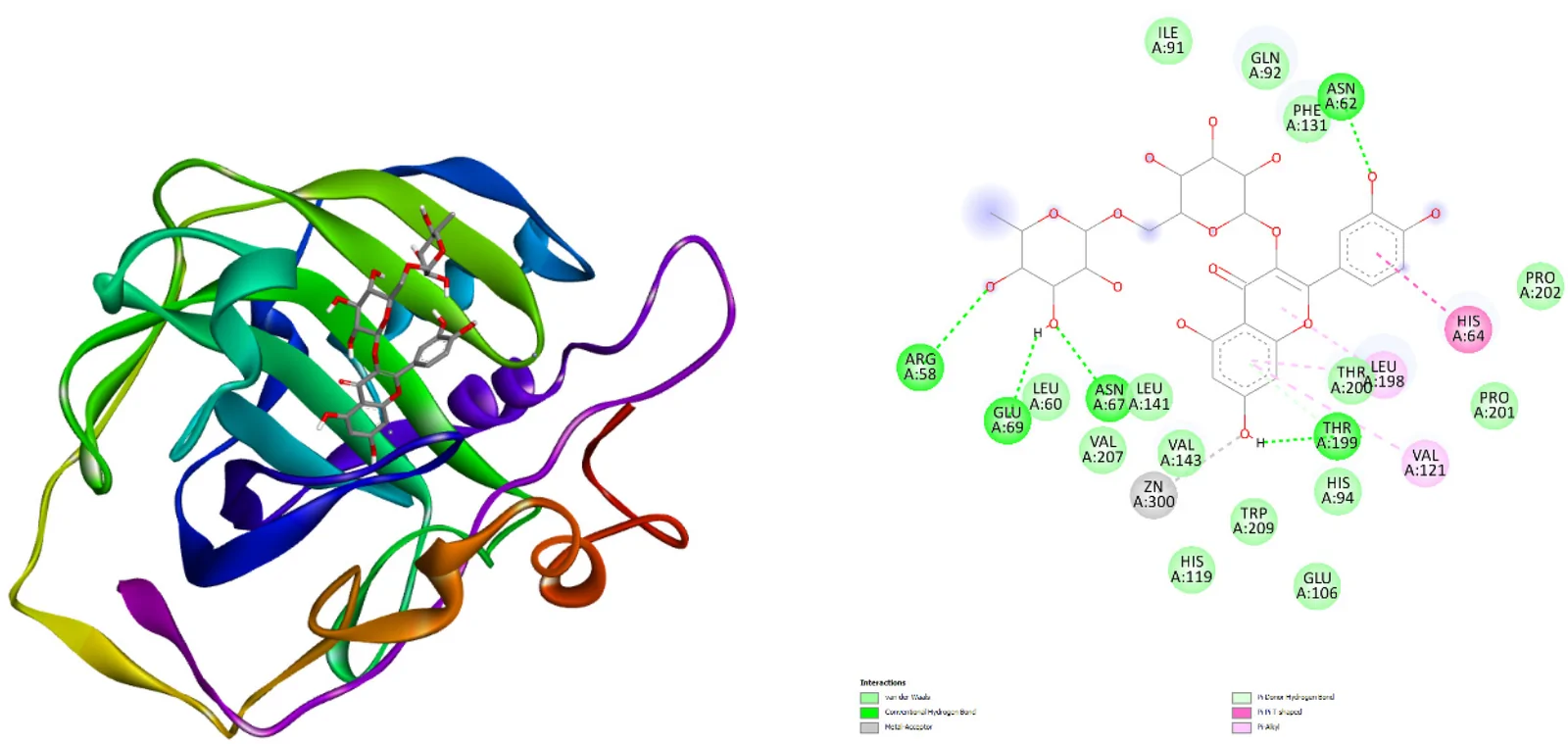
2D and 3D visualization of the docking results of rutin in the active site of carbonic anhydrase.

**Figure 16:**
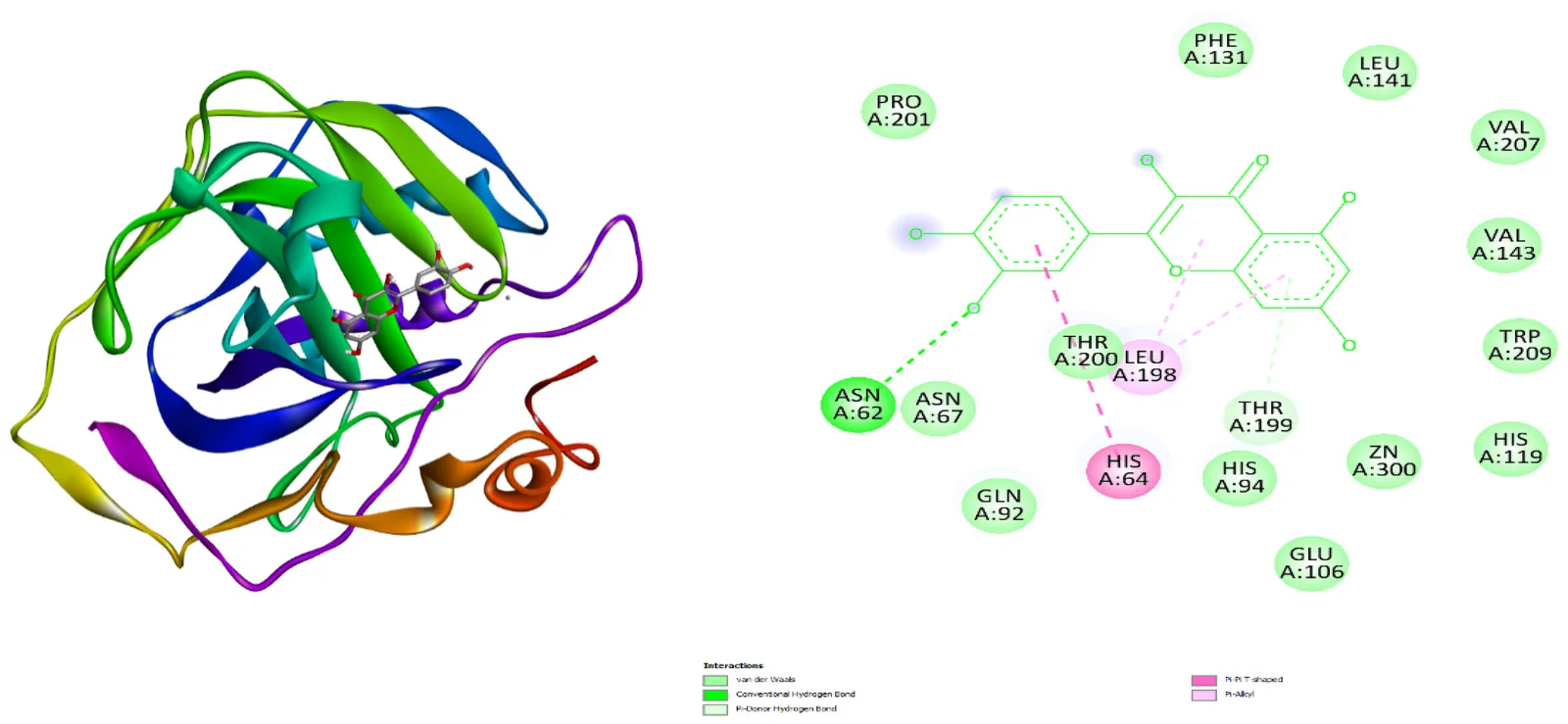
2D and 3D visualization of the docking results of quercetin in the active site of carbonic anhydrase.

Eight compounds were tentatively assigned in the HPLC analysis and subsequently considered for the ADME evaluation. For the molecular docking analysis, seven compounds were selected, while p-coumaric acid was not included in the docking workflow. The seven selected compounds were subjected to ligand preparation and molecular docking against carbonic anhydrase (Figure 10, Figure 11, Figure 12, Figure 13, Figure 14, Figure 15 and Figure 16).

The molecular docking results of seven compounds identified in the aqueous extract of *N. sativa* seeds with carbonic anhydrase revealed variable predicted binding affinities depending on the chemical nature of the tested molecules (Table 5). Rutin exhibited the most favorable predicted binding affinity, with a docking score of −8.6 kcal/mol, and formed several types of interactions, including conventional hydrogen bonds, Pi–alkyl interactions, Pi–Pi T-shaped interactions, Pi-donor hydrogen interactions, and a metal–acceptor interaction with residues within the enzyme active site. Quercetin also showed a favorable predicted binding affinity, with a docking score of −8.0 kcal/mol, and interacted with ASN-62, LEU-198, HIS-64, and THR-200 through conventional hydrogen bonds, Pi–alkyl, Pi–Pi T-shaped, and Pi-donor hydrogen interactions. Rosmarinic acid displayed a docking score of −7.4 kcal/mol and established several interactions, including conventional hydrogen bonds, Pi–alkyl, Pi-cation, and Pi-donor hydrogen interactions, together with one unfavorable donor–donor interaction.

The remaining compounds showed comparatively less favorable predicted binding affinities, with docking scores of −6.6 kcal/mol for caffeic acid, −6.2 kcal/mol for cinnamic acid, −6.0 kcal/mol for gallic acid, and −5.9 kcal/mol for syringic acid. Overall, rutin and quercetin exhibited the most favorable predicted interactions with the carbonic anhydrase active site among the compounds investigated. However, these computational findings should be considered predictive and do not establish carbonic anhydrase inhibitory activity in the absence of experimental enzyme-inhibition assays.

#### 2.4.5. Physicochemical and ADME Properties of *N. sativa* Compounds

Assessment of the physicochemical and pharmacokinetic properties of the eight compounds derived from *Nigella sativa* seeds provides essential clues as to their potential as oral drug candidates. According to Lipinski’s rule of five, widely used to assess drug-likeness, seven of the eight molecules met all the criteria, suggesting favorable predicted intestinal absorption. Only rutin (M6) breaks several rules because of its high molecular weight (610.52 g/mol), its excessive total polar surface area (269.43 Å^2^), and its large number of hydrogen bond donors and acceptors, which could limit its membrane permeability and absorption.

With regard to absorption, most of the compounds (Table 6), such as gallic acid (M1), caffeic acid (M2), syringic acid (M3), p-coumaric acid (M4), cinnamic acid (M5) and quercetin (M7), showed high predicted human intestinal absorption, confirming their good oral bioavailability. However, rutin (M6) and rosmarinic acid (M8) were poorly absorbed, probably due to their large size and high polarity.

Analysis of the permeability of the blood–brain barrier (BBB) indicates that only p-coumaric acid (M4) and cinnamic acid (M5) are likely to cross it, suggesting possible activity on the central nervous system. The other compounds show no potential for passage. About skin permeability (Log Kp), the values observed were low for all the compounds, particularly for rutin (M6), which had the lowest value (−10.26), reflecting very limited transdermal diffusion.

Metabolically, none of the compounds is a predicted substrate or major inhibitor of cytochrome P_450_ enzymes, thus reducing the risk of metabolic drug interactions. One exception is quercetin (M7), which is predicted to inhibit CYP3A4 and CYP1A2 and requires further in vivo evaluation. Rutin (M6) has also been identified as a substrate for P-glycoprotein, which could limit its intracellular accumulation via an active efflux mechanism.

Topological polar surface area (TPSA) values show that the majority of compounds are within the optimal range for oral administration (<140 Å^2^), with the exception of rutin (M6) and rosmarinic acid (M8), which may explain their poor absorption.

Water solubility (Log S) varied considerably between compounds, with cinnamic acid (M5) and gallic acid (M1) being the most soluble, while quercetin (M7) and rosmarinic acid (M8) showed low solubility, which could represent a challenge for their formulation.

In terms of drug-likeness, most compounds scored above 0.5 on the bioavailability scale, indicating good potential for systemic activity. However, rutin, with a low score (0.17), shows significant pharmacokinetic limitations despite its marked biological activity. In addition, the synthetic accessibility (SA) score, which ranges from 1 (easy to synthesise) to 10 (very difficult), reveals that compounds such as gallic acid (1.70) and caffeic acid (1.22) are easy to synthesise, while rutin (6.52) shows significant synthetic complexity (Table 7).

**Figure 17:**
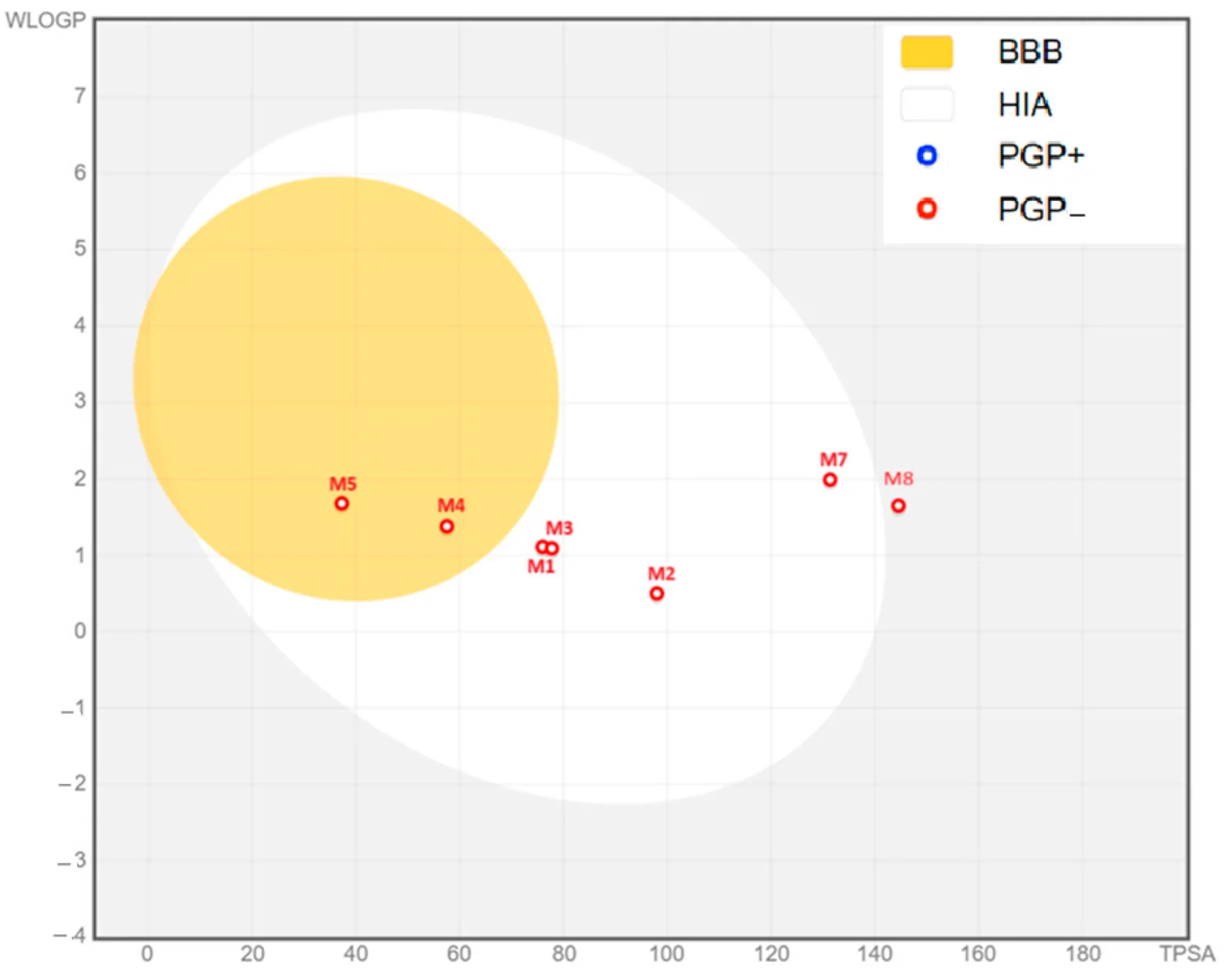
Predictive model of Egan’s boiled egg for 08 extracted molecules.

Finally, Egan’s “boiled egg” visual model (Figure 16) supports these results by classifying compounds according to their passive gastrointestinal absorption and their ability to penetrate the brain. Compounds in the white zone (HIA+) are likely to be well absorbed, while those in the yellow (BBB+) can also cross the blood–brain barrier. In this context, compounds such as cinnamic acid and p-coumaric acid appear in favourable zones, reinforcing their drug-likeness profile (Figure 17).

## 3. Discussion

The study conducted on the aqueous extract of *Nigella sativa* highlights a phytochemical profile rich in phenolic compounds and flavonoids, notably quercetin and rutin. These molecules are well known for their antioxidant and pharmacological properties, thereby reinforcing the potential therapeutic interest of this medicinal plant.

The evaluation of antioxidant activity, through various complementary tests (DPPH, reducing power, total antioxidant capacity), demonstrated that the aqueous extract possesses notable radical scavenging activity. Although this activity remains lower than that of standard antioxidants such as quercetin and BHT, the results show a dose-dependent effectiveness, with an inhibition rate of 68% in the DPPH test at the highest concentration, and an IC_50_ of 0.254 ± 0.002 mg/mL. The reducing power, with an EC_50_ of 0.247 ± 0.012 mg/mL, also indicates the extract’s ability to reduce oxidizing species. The total antioxidant capacity (TAC), particularly high (125.01 ± 4.220 mg AAE/g of extract), underlines the richness of the extract in water-soluble antioxidants, likely acting synergistically.

From a pharmacological perspective, the aqueous extract of *Nigella sativa* showed a measurable diuretic effect following oral administration at a dose of 300 mg/kg for 15 days. A significant increase in urinary sodium, potassium, chloride, and creatinine concentrations was observed. Although less pronounced than that of furosemide, this effect remains significant and biologically relevant. This pattern, together with the concurrent decrease in plasma electrolyte concentrations, is consistent with a diuretic/saliuretic effect; however, renal clearance was not calculated in this study and would be required to confirm quantitative changes in excretory function. Moreover, the slight hypokalemia, hyponatremia, and hypochloremia observed in plasma confirm increased urinary electrolyte loss, consistent with a saliuretic mechanism.

Regarding safety, the in vitro cytotoxicity study on primary immune cells (splenocytes and thymocytes) revealed excellent tolerance of the extract. No significant cytotoxicity was detected up to 1 mg/mL, with cell viability rates above 90%. A slight metabolic stimulation was observed, particularly in thymocytes, suggesting possible mitogenic or immunomodulatory activity.

This favorable profile can be attributed to the hydrophilic nature of the extract’s constituents, often correlated with better biocompatibility. Compared to other natural substances, *Nigella sativa* thus appears to offer an interesting combination of biological efficacy and low toxicity, making it promising for therapeutic applications, particularly in renal and cardiovascular disorders.

Several studies support these observations. *Nigella sativa* is recognized for its antihypertensive and nephroprotective properties, often attributed to its antioxidant activity and modulatory effects on oxidative stress, inflammation, and vascular signaling pathways [14]. Furthermore, its efficacy against nephrotoxicity induced by various chemical agents (carbon tetrachloride, cadmium chloride, paracetamol, etc.) has been demonstrated in animal models [12,15], through the reduction of lipid peroxidation and restoration of antioxidant enzyme activity (SOD, catalase).

However, despite its benefits, uncontrolled use of medicinal plants can lead to adverse effects. In Morocco, plant-induced nephrotoxicity is a public health issue due to poorly regulated empirical practices [16]. This highlights the importance of promoting rational and regulated use of these extracts, based on experimental and clinical evidence.

The aqueous extract of *Nigella sativa* possesses measurable antioxidant and diuretic potential, without notable cytotoxicity, justifying its exploration for applications in phytotherapy, particularly in renal, cardiovascular, and immune-related fields. These results support the need for further in vivo and clinical studies to better understand its mechanisms of action and ensure its long-term safety.

## 4. Materials and Methods

### 4.1. Preparation of Plant Material

*Nigella sativa* (black cumin) seeds used in this study were purchased from a local herbalist and originated from the Souk El Arbaa region of Morocco (34°39′57″ N, 5°58′54″ W). The samples were ground into a fine powder and stored in airtight glass containers, protected from light and moisture, before analysis.

#### 4.1.1. Preparation of Aqueous Extracts

A quantity of 5 mg of cleaned and ground *N. sativa* seed powder was macerated in 10 mL of distilled water under gentle agitation overnight at ambient temperature. After filtration using filter paper, the aqueous extract was collected. The solvent was then evaporated under reduced pressure using a rotary evaporator (R-300, Büchi Labortechnik AG, Flawil, Switzerland), yielding a dark-colored crude aqueous extract.

#### 4.1.2. Determination of Total Phenolic Content

The total phenolic content was determined using the Folin–Ciocalteu method as described by [17]. Briefly, 200 µL of extract was mixed with 1 mL of Folin–Ciocalteu reagent (diluted 1:10), followed by the addition of 0.8 mL of 7.5% sodium carbonate (Na_2_CO_3_). The mixture was incubated at room temperature for 30 min, and absorbance was measured at 765 nm using a UV-Vis spectrophotometer. Results were expressed as milligrams of gallic acid equivalents (mg GAE) per gram of dry plant material.

#### 4.1.3. Determination of Total Flavonoid Content (TFC)

The total flavonoid content of the aqueous extract was assessed using the aluminum chloride colorimetric method, which is widely employed to quantify flavonoids in plant extracts [18]. In brief, 0.5 mL of the extract was mixed with 0.1 mL of 10% aluminum chloride, 0.1 mL of 1 M potassium acetate, 1.5 mL of 80% methanol, and 2.8 mL of distilled water. After incubation at room temperature for 30 min, absorbance was recorded at 415 nm. A blank was prepared under the same conditions, and quercetin was used as the standard. Results were expressed as milligrams of quercetin equivalents (mg QE) per gram of extract.

#### 4.1.4. The Total Carbohydrate Content

A volume of 200 µL of each extract was mixed with 500 µL of concentrated sulfuric acid (96–98% *v*/*v*), followed by the addition of 30 µL of 5% phenol reagent (0.2 N) [19]. The mixture was heated for 5 min at 90 °C and then allowed to cool to room temperature for another 5 min. Absorbance was measured at 490 nm. A calibration curve was established using glucose standards ranging from 10 to 600 mg/L (R^2^ = 0.992). The results were expressed as grams of glucose equivalent per 100 g of dry plant material (g GluE/100 g DPM).

#### 4.1.5. HPLC-DAD Analysis

The aqueous extract of *N. sativa* and the standard reference compounds were dissolved at a concentration of 30 mg/mL and filtered through 0.4 µm membrane filters to remove particulate matter prior to chromatographic analysis. Separation was performed using a reversed-phase Kinetex C18 column (250 × 4.6 mm, 2.6 µm particle size) on an HPLC system equipped with a diode-array detector (HPLC-DAD), following chromatographic conditions previously applied in studies of *Nigella sativa*. A binary gradient consisting of 0.1% acetic acid in water (solvent A) and methanol (solvent B) was used: from 0 to 3 min, B increased from 5 to 25%; 3–6 min, isocratic at 25% B; 6–9 min, from 25 to 37% B; 9–13 min, isocratic at 37% B; 13–18 min, from 37 to 54% B; 18–22 min, isocratic at 54% B; 22–26 min, from 54 to 95% B; 26–29 min, isocratic at 95% B; 29–35 min, from 95 to 5% B; and 35–45 min, isocratic at 5% B. The flow rate was maintained at 1 mL/min, and the column temperature was set at 30 °C. Detection was performed over the spectral range of 200–400 nm, with chromatograms monitored at 280 nm for optimal detection of the phenolic compounds. Compounds were tentatively assigned by comparing the retention times of sample peaks with those of authentic reference standards analyzed under the same chromatographic conditions [20]. Accordingly, the HPLC-DAD results were considered tentative assignments rather than definitive compound identification.

### 4.2. Biological Activity of the Aqueous Extract of Nigella sativa Seeds

The biological activities of the aqueous extract of *N. sativa* seeds were evaluated through various in silico, in vitro, and in vivo assays.

#### 4.2.1. Antioxidant Activity

The antioxidant potential of the aqueous extract was assessed using three complementary in vitro methods: DPPH radical scavenging, Total Antioxidant Capacity (TAC), and Ferric Reducing Antioxidant Power (FRAP).

##### DPPH Radical Scavenging

The ability of the extract to scavenge the stable DPPH radical was evaluated. A 50 µL aliquot of the extract at various concentrations was added to 850 µL of a 4 mg/100 mL DPPH solution. After 20 min of incubation in the dark at room temperature, the absorbance was measured at 517 nm. The percentage inhibition was calculated using the formula [21]:

$$\%Inhibition=\left(\frac{Ac-Ae}{Ac}\right)\times 100$$

where Ac is the absorbance of the control, and Ae is the absorbance of the extract. Quercetin and BHT were used as reference antioxidants.

##### Total Antioxidant Capacity (TAC)

The assay is based on the reduction of molybdate (VI) to molybdate (V) in an acidic medium, forming a green phosphomolybdic complex [21]. A 200 µL aliquot of the extract was mixed with 2 mL of a reagent solution containing 0.6 mol/L sulfuric acid, 28 mmol/L sodium phosphate, and 4 mmol/L ammonium molybdate. The mixture was incubated at 95 °C for 90 min, cooled to room temperature, and the absorbance was measured at 695 nm. The antioxidant activity was expressed as milligrams of ascorbic acid equivalent per gram of extract (mg EAA/g).

##### Ferric Reducing Antioxidant Power (FRAP)

The reducing power of the extract was determined by mixing 200 µL of the aqueous extract with 500 µL of 0.2 M phosphate buffer (pH 6.6) and 500 µL of 1% potassium ferricyanide [15]. The mixture was incubated at 50 °C for 20 min, followed by the addition of 500 µL of 10% trichloroacetic acid to stop the reaction. After centrifugation, 500 µL of the supernatant was mixed with 100 µL of 0.1% FeCl_3_ and 500 µL of distilled water. The absorbance of the resulting complex was measured at 700 nm [21].

#### 4.2.2. Evaluation of Cell Proliferation by MTT Assay Cell Culture

##### Cell Culture

For this study, cell suspensions were obtained from six healthy adult male New Zealand White rabbits, aged 10–12 weeks and weighing 2 ± 0.2 kg, obtained from the animal facility of the Faculty of Sciences and Technology. Deep inhalation anesthesia was induced using pharmaceutical-grade diethyl ether in an induction chamber until complete loss of consciousness and pedal reflexes was established. Euthanasia was then humanely performed via cervical dislocation. All animal experimental procedures complied with ethical guidelines for laboratory animal care, following the Guide for the Care and Use of Laboratory Animals (National Research Council, 2011), and were approved by the Ethics Committee of the Faculty of Science and Technology (CEFST) under reference number 16/2022/CEFST.

Lymphoid organs (spleen and thymus) were aseptically collected. Cell suspensions were prepared by mechanical dissociation of the tissues using a fine mesh sieve, as previously described by [22,23]. The obtained cells were washed with RPMI medium, and erythrocytes were removed using a 154 mM ammonium chloride solution. Cell viability was assessed using the trypan blue exclusion method (0.1%) and observed under an optical microscope. Cultures were maintained in RPMI medium supplemented with glutamine (2 mM), fetal bovine serum (10%), antibiotics (ampicillin 100 U/mL, streptomycin 100 µg/mL), and an antifungal agent (fluconazole µg/mL), to ensure optimal sterile conditions for cell growth.

##### Cell Proliferation Assay

Cell proliferation was evaluated using the colorimetric MTT assay, according to the original protocol of [20], with slight modifications [16,17,18]. Isolated cells (2 × 10^5^ cells/well) were cultured in 96-well plates and incubated with various concentrations (0.1–1 mg/mL) of the extract diluted in complete RPMI medium. Each experimental condition was tested using cells isolated from six individual animals (*n* = 6 independent biological replicates), with each sample run in triplicate wells (*n* = 3 technical replicates). Incubation lasted for 72 h at 37 °C in a humidified atmosphere containing 5% CO_2_. At the end of the incubation period, 10 µL of MTT solution (5 mg/mL in PBS) was added to each well. After three hours of reaction, DMSO was added to solubilize the formazan crystals formed, which indicate the metabolic activity of viable cells. Absorbance was measured at 450 nm using a microplate reader (BioTek L800), thereby allowing the quantification of cell proliferation.

#### 4.2.3. Ethical Approval

The study was carried out on healthy male albino rats, each weighing between 180 and 250 g, obtained from the animal facility of the Faculty of Science at Sidi Mohamed Ben Abdellah University in Fez, Morocco. The animals were housed under standard laboratory conditions, with a controlled temperature of 25 ± 1 °C and a 12 h light/dark cycle. They had free access to tap water and standard laboratory chow ad libitum. All procedures involving animals were conducted in strict accordance with international, national, and institutional guidelines for the care and use of laboratory animals. The experimental protocol was reviewed and approved by the Institutional Ethics Committee of the Faculty of Science, Sidi Mohamed Ben Abdellah University (Reference: L.20. USMBA-SNAMOPEQ 2023-03). Rats were humanely euthanized by an overdose of pentobarbital in accordance with the ARRIVE guidelines (Animal Research: Reporting of In Vivo Experiments). The plasma samples were collected by retro-orbital collection and transferred into heparinized capillary tubes under light anesthesia with sodium pentobarbital, which was given through intraperitoneal injections of 30 mg/kg [11].

#### 4.2.4. Diuretic Activity

##### Experimental Design

Prior to the start of the experiment, each rat was placed in a metabolic cage for 48 h to acclimate to the experimental conditions. The animals were then divided into three groups, each consisting of four rats:

- **Group 1 (negative control):** received distilled water (10 mL/kg body weight),
- **Group 2 (positive control):** received furosemide orally (10 mg/kg body weight),
- **Group 3:** received the aqueous extract of *Nigella sativa* seeds at a dose of 300 mg/kg.

Treatments were administered orally once daily for a period of 15 days.

##### Urine and Blood Collection and Analysis

Urinary excretion was assessed on days 1, 7, and 15 of treatment. Urine was collected in graduated cylinders to measure the excreted volumes. Subsequently, analyses were performed to determine the concentrations of sodium, potassium, chlorine, and creatinine. Urine samples were stored at −20 °C until analysis.

### 4.3. In Silico Study

#### 4.3.1. Molecular Modeling of Different Compounds from the Aqueous Extract of *N. sativa* Seeds in Interaction with Carbonic Anhydrase

##### Ligand Preparation

The three-dimensional structures of various compounds from the aqueous extract of *N. sativa* seeds were obtained from the PubChem database [23] in SDF format. These compounds included gallic acid (CID: 370), caffeic acid (CID: 689043), syringic acid (CID: 10742), cinnamic acid (CID: 444539), rosmarinic acid (CID: 5281792), rutin (CID: 5280805), and quercetin (CID: 5280343).

These molecules were then converted into PDB format using PyMOL 3.1 software. Molecular configurations were prepared using AutoDockTools version 1.5.6 and saved in PDBQT format for subsequent in silico analysis.

##### Protein Preparation

The X-ray crystallographic structures were retrieved from the RCSB Protein Data Bank (PDB) [24]. For the molecular docking study, the selected target protein was carbonic anhydrase (PDB ID: 1Z9Y). Preparation of the protein was carried out using AutoDockTools by removing water molecules, adding Kollman charges and polar hydrogens. The processed structure was then converted and saved in PDBQT format for molecular docking simulations.

##### Molecular Docking

Molecular docking was performed using AutoDock Vina to estimate the predicted binding affinity of the ligands to their protein targets, expressed in kcal/mol. The most stable ligand conformation corresponds to the one with the lowest predicted binding affinity, indicating a favorable interaction with the protein’s active site [12]. The grid box was defined by centering the coordinates on the active site of the target protein (x = 11.742066, y = 0.013043, z = 16.242960). The dimensions of the grid were set to 40 Å × 40 Å × 40 Å, with a spacing of 0.375 Å. The docking poses and visualization of 2D and 3D ligand-receptor interactions were analyzed using Discovery Studio software. The crystallographic furosemide, a sulfonamide inhibitor, was redocked into the active site of carbonic anhydrase II (PDB ID: 1Z9Y) to validate the molecular docking protocol. PyMOL software was used to calculate the root-mean-square deviation (RMSD) between the experimentally determined crystallographic pose of furosemide and its redocked pose. According to Olejarz et al. (2025) [25], an RMSD value ≤ 3.0 Å indicates good agreement between the experimentally determined and predicted binding poses, thereby supporting the reliability of the docking protocol.

##### Physicochemical Properties, Pharmacokinetic ADME Profiling

An in silico analysis of the bioactive compounds extracted from *N. sativa* seeds was conducted to assess their compliance with Lipinski’s Rule of Five, their pharmacokinetic properties (ADME), and their potential for molecular interactions. These predictions help evaluate the “drug-likeness” of each compound—that is, its likelihood of becoming an orally administered drug.

### 4.4. Statistical Analysis

The results of the three independent experiments were expressed as mean values accompanied by standard deviation (SD). Prior to statistical testing, data were assessed for normality and homogeneity of variances to determine the appropriate analysis approach (parametric or non-parametric). The significance of differences between means was evaluated using one-way and two-way analysis of variance (ANOVA). Post hoc comparisons were carried out using Tukey’s multiple comparison test. All statistical analyses were performed using GraphPad Prism version 8.0.2, with a significance threshold set at *p* ≤ 0.05.

## 5. Conclusions

The results of this study highlight the pharmacological potential of the aqueous extract of *Nigella sativa*, particularly its antioxidant activity, significant diuretic effect, and good cytotoxic tolerance. Thanks to its richness in phenolic compounds and flavonoids, the extract showed an interesting ability to scavenge free radicals, reduce pro-oxidant metals, and maintain a high total antioxidant capacity. the extract induced diuresis and changed the plasma and urinary electrolyte and creatinine levels, while showing no notable toxicity on tested immune cells.

These results support the traditional uses of *Nigella sativa* and reinforce its potential in phytotherapy for the prevention or management of conditions related to oxidative stress, fluid retention, or hypertension. However, despite the observed effectiveness, the extract remains less potent than some reference compounds, highlighting the importance of continuing investigations.

## Figures and Tables

**Table 1:** Total contents of phenolic compounds (TPC), flavonoids (TFC), and carbohydrates.

Aqueous Extract	TPC (mg GAE/g D.W.)	TFC (mg QE/g D.W.)	Carbohydrates (g GluE/100 g D.W.)
	22.88 ± 1.12	19.14 ± 1.31	5.817 ± 0.04
			5.817

GAE: Gallic Acid Equivalent; QE: Quercetin equivalent; GluE: Glucose Equivalent; D.W: Dry Weight.

**Table 2:** Phenolic compounds tentatively assigned by HPLC-DAD *N. sativa* extract and their relative chromatographic peak areas.

Compound	Retention Time (Min)	Relative Peak Area (%)
**Gallic acid**	5.45	05.77
**Caffeic acid**	11.55	00.49
**Syringic acid**	11.98	00.31
***p*** **-Coumaric acid**	14.68	13.19
**Cinnamic acid**	22.10	02.33
**Rutin**	27.28	14.06
**Quercetin**	30.38	29.58
**Rosmarinic acid**	31.60	4.92

Area (%) values represent relative chromatographic abundance and do not correspond to absolute concentrations or contents of the compounds in the extract.

**Table 3:** Antioxidant activity results of the aqueous extract of *N. sativa*.

Sample	DPPH IC_50_ (mg/mL)	FRAP EC_50_ (mg/mL)	TAC (mg AAE/g Extract)
***N. sativa***	0.254 ± 0.002	0.247 ± 0.012	125.01 ± 4.220
**BHT**	0.012 ± 0.001	0.139 ± 0.011	47.28 ± 1.050
**Quercetin**	0.035 ± 0.040	0.041 ± 0.020	27.81 ± 0.981

**Table 4:** Urine volume (mL/animal) over the 15-day treatment period, mean ± SD.

Day	Control	Furosemide, 10 mg/kg	*N. sativa*, 300 mg/kg
1	3.75 ± 0.06	7.05 ± 0.06	8.55 ± 0.06
7	4.13 ± 0.38	9.10 ± 1.04	10.07 ± 0.90
15	4.60 ± 0.69	13.20 ± 1.15	13.67 ± 0.58

**Table 5:** Binding energies of compounds with the active site of carbonic anhydrase.

Compounds	PubChem ID	Binding Affinity (kcal/mol)	Type of Interactions
Gallic acid	370	−6.0	Conventional hydrogen bonds, Pi–Pi stacked, and unfavorable Donor–Donor interaction
Caffeic acid	689043	−6.6	Conventional hydrogen bonds, Pi–alkyl, and unfavorable Donor–Donor interaction
Syringic acid	10742	−5.9	Conventional hydrogen bonds, alkyl, Pi–alkyl, Pi–Pi stacked, Pi–sigma, carbon–hydrogen bond, and unfavorable Donor–Donor interaction
Cinnamic acid	444539	−6.2	Conventional hydrogen bonds, Pi–alkyl, Pi–Pi stacked, and unfavorable Donor–Donor interaction
Rosmarinic acid	5281792	−7.4	Conventional hydrogen bonds, Pi–alkyl, Pi–cation, Pi–Donor hydrogen bond, and unfavorable Donor–Donor interaction
Rutin	5280805	−8.6	Conventional hydrogen bonds, Pi–alkyl, Pi–Pi T-shaped, Pi–Donor hydrogen bond, and metal–acceptor interaction
Quercetin	5280343	−8.0	Conventional hydrogen bonds, Pi–alkyl, Pi–Pi T-shaped, and Pi–Donor hydrogen bond

**Table 6:** Prediction of physicochemical properties of 08 chemical compounds extracted from *Nigella sativa* seeds based on Lipinski’s rule of five.

	Physicochemical Properties					Lipinski Rules
	*Molecular* *Weight (g/mol)*	*Molar* *Refractive* *Index*	*Log P* *(Octanol/* *Water)*	*Hydrogen Bond* *Acceptors*	*Hydrogen Bond* *Donors*	*Categorical* *(Yes/No)*
Rule	≤500	40≤ MR ≤ 130	<5	≤10	<5	
M1	170.12	39.47	0.21	5	4	Yes
M2	180.16	47.16	0.97	4	3	Yes
M3	198.17	48.41	1.54	5	2	Yes
M4	164.16	45.13	0.95	3	2	Yes
M5	148.16	43.11	1.55	2	1	Yes
M6	610.52	141.38	0.46	16	10	No
M7	302.24	78.03	1.63	7	5	Yes
M8	360.31	91.40	1.48	8	5	Yes

M1: Gallic acid; M2: Caffeic acid; M3: Syringic acid; M4: p-Coumaric acid; M5: Cinnamic acid; M6: Rutin; M7: Quercetin; M8: Rosmarinic acid.

**Table 7:** Prediction of ADME pharmacokinetic properties of 08 compounds extracted from *Nigella sativa* seeds.

Molecule Number	Absorption	Distribution		Metabolism						Physicochemical Properties		Druglikeness	Medicinal Chemistry
	Human Intestinal Absorption	Blood–Brain Barrier Permeant	Skin Permeation	Substrate	Inhibitor					Topological Polar Surface Area	Water Solubility	Bioavailability Score	Synthetic Accessibility
				Cytochromes									
				P-glycoprotein	CYP1 A2	CYP2C19	CYP2C9	CYP2D6	CYP3A4				
	(Absorbed)	(No/Yes)	(Log *K*_p_)	(No/Yes)						(Å^2^)	Log *S* (SILICOS-IT)		
M1	High	No	−6.77	No	No	No	No	No	No	75.99	−1.46	0.56	1.70
M2	High	No	−6.84	No	No	No	No	No	Yes	97.99	−0.04	0.56	1.22
M3	High	No	−6.58	No	No	No	No	No	No	77.76	−0.71	0.56	1.81
M4	High	Yes	−6.26	No	No	No	No	No	No	57.53	−1.28	0.85	1.61
M5	High	Yes	−5.69	No	No	No	No	No	No	37.30	−1.84	0.85	1.67
M6	Low	No	−10.26	Yes	No	No	No	No	No	269.43	−0.29	0.17	6.52
M7	High	No	−7.05	No	Yes	No	No	Yes	Yes	131.36	−3.24	0.55	3.23
M8	Low	No	−6.82	No	No	No	No	No	No	144.52	−2.17	0.56	3.38

M1: Gallic acid; M2: Caffeic acid; M3: Syringic acid; M4: p-Coumaric acid; M5: Cinnamic acid; M6: Rutin; M7: Quercetin; M8: Rosmarinic acid.

## Data Availability

The original contributions presented in this study are included in the article. Further inquiries can be directed to the corresponding authors.
